# Stress Concentration Factors for Non-Load-Carrying Welded Cruciform Joints Subjected to Tension, Bending, and Shear

**DOI:** 10.3390/ma17030609

**Published:** 2024-01-26

**Authors:** Krzysztof L. Molski, Piotr Tarasiuk

**Affiliations:** 1 SaMASZ Sp. z o.o., ul. Trawiasta 1, 16-060 Zabludow, Poland; krzysztof.molski@samasz.pl; 2 Faculty of Mechanical Engineering, Bialystok University of Technology, Wiejska 45C, 15-351 Bialystok, Poland

**Keywords:** welded plate cruciform connections, stress concentration factor, weld toe, finite element analysis, tension, bending, shear

## Abstract

This paper deals with the problem of stress concentration at the weld toe of non-load-carrying-type plate cruciform joints under tension, bending, and shear. Theoretical stress concentration factors were derived using the finite element method. Five of the most important geometrical parameters: the thickness of the main plate and the attachments, the weld throat thickness, the weld toe radius, and the weld face inclination angle were treated as independent variables. For each loading mode—tension, bending, and shear—parametric expression of high accuracy was obtained, covering the range used in real structures for cruciform connections. The maximum percentage error was lower than 2.5% as compared to numerical values. The presented solutions proved to be valid for the toe radius *ρ* tending to zero.

## 1. Introduction

Welded cruciform joints are commonly used in real welded structures. Two types of such connections are generally distinguished: a load-carrying one and a non-load-carrying one, depending on the way the external load is transmitted through the joint. In the former case, the loaded plates are connected to the transversal one, and the whole load passes through the welds. In such a case, so-called lack of penetration defects may occur in the form of crack-like transversal flaws [1,2,3,4]. In the latter case, the main plate can sustain the whole load while additional attachments are welded to both sides of the main plate.

It is well known that the fatigue fracture of welded connections subjected to variable loads is the most common type of damage [5,6,7,8], which usually begins at critical points related to the maximum stress concentration. During the last few decades, a great effort has been made in analyzing fatigue phenomena. However, the problem of efficient design procedures has not been definitely solved and many research programs are currently being developed to further address this issue.

Various approaches are used to assess the strength and durability of weldments. Some are based on local concepts including hot spot, local stress [9,10,11,12]. and fracture mechanics [13,14,15].

It is generally accepted that the geometry of a joint has a significant influence on the fatigue strength and durability of the welded structure. In particular, the weld toe radius *ρ*, the weld angle *θ*, and the bead profile are of primary importance [2,8,13,16,17,18,19]. Unfortunately, all of these parameters change in a random way along the weld seam. Some examples of statistical data may be found in [16,20]. In spite of all of these difficulties, various standards and recommendations, presented in References [21,22,23,24,25,26], have been developed, which have proven to be significantly helpful in the fatigue design of welded structures, including cruciform connections.

The mechanisms of fatigue damage are very specific and governed by the stress range Δσ and the stress gradient at the critical point of a cruciform joint, which depends mainly on the weld toe radius or the size and shape of additional imperfections that may appear in a joint. The geometry of the joint is the key quantity because it determines the SCF values. There are several important arguments confirming this conclusion:

- In EN ISO 5817:2014 [27], quality levels for imperfections are described. Numerous geometrical cases of imperfections are depicted in the form of tables. All geometrical cases do not depend on the material properties and are valid for all specified materials.
- Many recommended mechanical methods applied to improve the fatigue strength of weldments, such as grinding, re-melting, weld reinforcement, various types of hammer-peening, etc., consist of changing the weld toe geometry by increasing the radius to reduce the stress concentration, i.e., to diminish SCFs (see [27] Welding and allied processes. Quality levels for imperfections and the report of IIW Commission XIII, Recommendations on Post Weld Improvements of Steel and Aluminium Structure, Section 1: ‘Modification of weld toe geometry’).
- There are many papers relating the FAT approach to the particular types of welded joints. ‘Particular joints’ means ‘joints of different geometries’ and the ‘FAT approach’ means ‘fatigue life characteristics based on the variable stress range’. The conclusions are obvious. All remaining properties and factors are less important than the geometrical and loading ones.

The theoretical stress concentration factor (SCF) is one of the crucial parameters used in the design of weldments, as it enables relating the remote stresses to the local critical points. Some methods, applications, particular values, and formulas used for calculating SCFs are given in References [17,28,29,30,31,32,33,34,35].

From the analysis of the data available in the literature, one may conclude that there is a lack of information about the stress concentration in welded joints. Such data would be very helpful for designers in predicting the fatigue strength and life of welded structures including cruciform joints. As shown in [32], some existing formulas have generally undetermined accuracy and a relatively narrow range of application. Moreover, there are no solutions dealing with shearing loads.

The present work deals with the determination of stress concentration factors for non-load-carrying welded plate cruciform joints subjected to tension, bending, and shear, using the finite element method (FEM) and represented by appropriate parametric equations. Some SCF solutions have already been obtained earlier for welded cruciform connections [31] subjected to axial and bending loads, for *θ* = 45°. In the present work, some extended solutions are given for the weld angle *θ* changing from 30° to 60° and for the shearing load.

## 2. Methodology

### 2.1. General Assumptions

**Figure 1:**
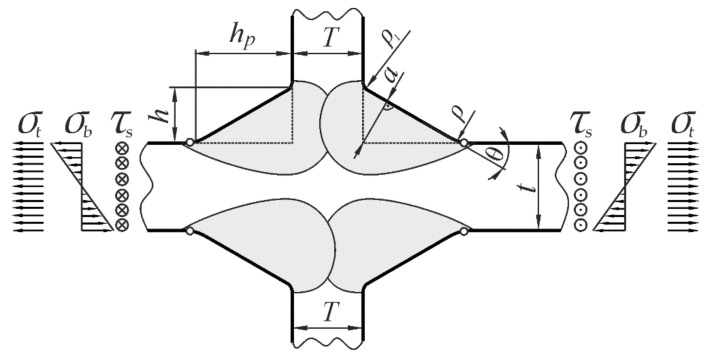
A cruciform welded joint, its geometrical parameters, and loading conditions.

The shape and geometrical dimensions of a cruciform joint and the three loading modes considered in the present work are depicted schematically in Figure 1. The location of maximum stresses is indicated by small circles.

Five geometrical parameters—*ρ*, *a*, *θ*, *t*, and *T*—influencing the SCF value and shown in Figure 1 were selected. Leg lengths, *h* and *h_p_*, characterizing the weld size, are also used in the literature.

In the present analysis, the following was assumed:

1. The material of the joint is linear elastic, homogeneous, and isotropic;
2. The elastic properties of the main plate, attachment plates, and welds are the same;
3. The welded joint is free from residual stresses, structural irregularities, and imperfections, including a lack of penetration defects;
4. Both attached plates are co-linear and perpendicular to the main plate;
5. All four welds satisfy the double symmetry of the connection;
6. The weld faces are plane, and the contour of the weldment is smooth with a transition toe radius *ρ* > 0;
7. External load—axial, bending, and shearing—is applied far enough from the welds in order to satisfy the principle of Saint-Venant;
8. Small deformations occur in the whole body;
9. Five geometrical parameters: *ρ*, *a*, *θ*, *t*, and *T* vary in the following ranges: 0 < *ρ*/*a* ≤ 1.3, 0 < *a*/*t* ≤ 1.3, 1 ≤ *T*/*a* ≤ 4, and 30° ≤ *θ* ≤ 60°;
10. Particular stress concentration factors for different loading modes are defined as follows: *K_tt_* = *σ*_1max_/*σ_t_*, *K_tb_* = *σ*_1max_/*σ_b_*, and *K_ts_* = *τ*_max_/*τ_s_*.

Any deviation from these assumptions, including the material properties, the shape of the body, and the loading conditions may cause significant changes in the stress distribution, including the maximum stress value at the critical point.

The aim of this work was to derive SCF parametric equations covering all possible values of five geometrical parameters characterising the cruciform joint. For this reason, a wide margin for the normalized parameters was used.

### 2.2. General Approach

The procedure of deriving three parametric formulae consists of several steps described in detail in Refs. [34,35]. The difference between a modeling welded T-joint and a cruciform connection lays only in the boundary conditions considered in the numerical model. Therefore, only some general steps of the procedure are mentioned in the present study.

First, conveniently defined new geometrical parameters are specified as follows:

(1) $$X=\rho /\left(\rho +a\right)=\frac{\rho /a}{\rho /a+1}$$

(2) $$Y=a/\left(a+t\right)=\frac{a/t}{a/t+1}$$

(3) Z=T/a

In this way, five independent parameters are converted into four variables *X*, *Y*, *Z*, and *θ*.

The second step consists of numerical modeling of the cruciform joint using the finite element method, where particular values of the four variables *X*, *Y*, *Z*, and *θ* systematically change. In this way, several thousand numerical solutions of SCF values are obtained. The third step of the approach required choosing a general form of the mathematical representation of the SCFs’ approximating functions. The general form of expressions and procedures used in the present article is similar to that presented in Reference [35] dealing with welded T-joints.

Hence, a general form of the approximating function is given by Equation (4):

(4) $$K_{t}=X^{n}P\left(X,Y,\theta ,Z_{0}\right)\kappa \left(X,Y,\theta ,Z_{0}\right)$$

where a singular term $X^{n}$ accounts for the stress concentration effects when *ρ*→0. The function *P*(*X*, *Y*, *θ*, *Z*_0_) is represented by polynomials and can be derived from numerical SCF solutions normalized with respect to the singular term, $K_{t}/X^{n}$ for different *X*, *Y*, and *θ*, while *Z* = *Z*_0_. From Equation (4), one may conclude that the *P* function cannot directly relate the SCF to *Z* because it is derived for a particular arbitrarily chosen value of *Z* = *Z*_0_. Therefore, the correction function *κ*(*X*, *Y*, *Z*, *θ*, *Z*_0_) must satisfy Equation (5):

(5) $$\kappa \left(X,Y,\theta ,Z_{0}\right)=1$$

As shown in [34,35], the exponent *n*, valid for tension and bending, and denoted as *n_s_* for shearing load, is necessary to describe the stress concentration effects when the weld toe radius tends to zero. The numerical values of the exponent *n*, for tension and bending, are given by Equation (6):

(6) $$n=\frac{-0.63662\theta -0.09330\theta^{2}}{1+0.77635\theta +0.04075\theta^{1.5}-0.00499\theta^{2}+0.13365\theta^{2.5}}\;$$

which is valid in the range of 0 ≤ *θ* ≤ π/2 with an accuracy of five significant digits.

In the case of anti-plane deformation, produced by the shearing load, the exact value of the exponent *n*_s_ equals:

(7) $$n_{s}=\frac{-\theta }{\theta +\pi }\;$$

where *θ* is in radians.

The next step of the procedure consists f approximating the normalized numerical SCF data with polynomial functions *P*(*X*,*Y*,*Z*_0_,*θ*) using the least-squares method. Next, the approximation accuracy of the *P* function is verified.

The last step of the present procedure consists of deriving the unknown coefficients and exponents of the correction function *κ*(*X*,*Y*,*Z*,*θ*,*Z*_0_). After performing the second validation of the full solution including *κ*, the close form approximating functions for calculating the SCFs were determined. The procedure was consecutively repeated for each loading mode. Approximating functions, in the form of parametric equations, are given in Appendix A.

## 3. Numerical FEM Modeling and Some SCF Results

### 3.1. Tensile and Bending Load

The ANSYS 19 Multiphysics FEM program was used for the systematic numerical modeling of cruciform joints. Four-node PLANE182 finite element, having 2 degrees of freedom at each node, was used in modeling. The elastic material constants: E = 210 GPa and ν = 0.3 were used.

**Figure 2:**
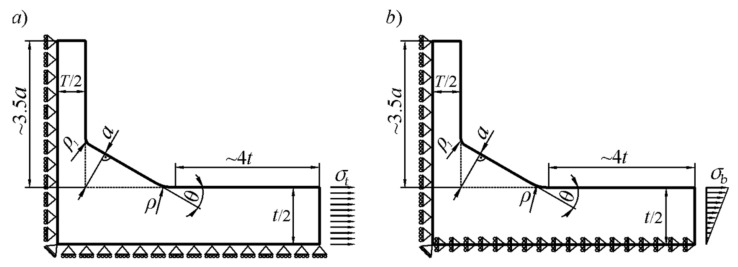
Boundary conditions used in the FEM model for tensile (**a**) and bending (**b**) loads.

The loading and displacement boundary conditions imposed on one quarter of the joint are shown in Figure 2. Considering the principle of Saint-Venant, the minimum length of the main plate measured from the weld toe was taken as 4*t*. This value was chosen by conducting preliminary numerical tests.

Before solving any FEM model, the finite element mesh density has to be found. This is usually carried out by solving the same problem using different numbers of elements and analyzing the subsequent results. This procedure was applied in the present case considering an increase in the mesh density in particular zones where the stress concentration occurs. For this reason, the dimensional ratio of neighboring finite elements was about 1.2, and approximately 40 to 70 finite elements were used along the weld toe circular arc. Approximately 730,000 finite elements were used for each geometrical case.

**Figure 3:**
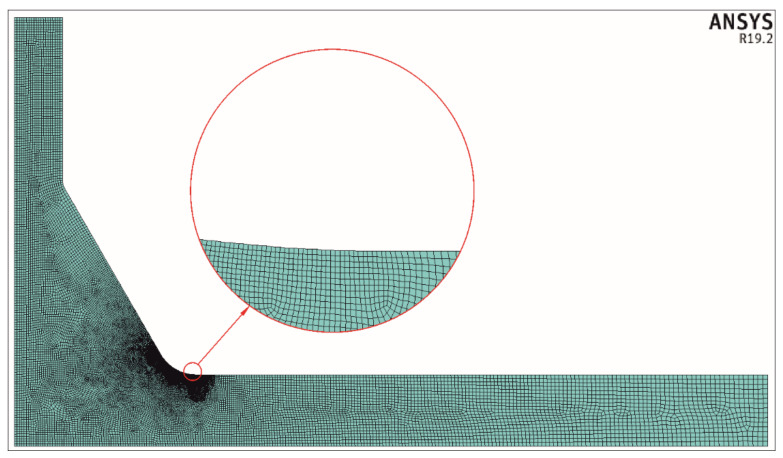
Example of a finite element mesh for *X* = 0.3, *Y* = 0.4, *θ* = 60°, and *T*/*a* = 1.

Considering the fact that the geometry of the joint changed significantly and a great number of FEM solutions had to be obtained, automation in generating finite element meshes was necessary. Therefore, some unique meshing algorithms were applied. One example of a finite element mesh is shown in Figure 3.

The first principal stresses *σ*_1_ are the most convenient values in deriving SCFs, because they are directly accessible, while nominal loads equal one.

**Figure 4:**
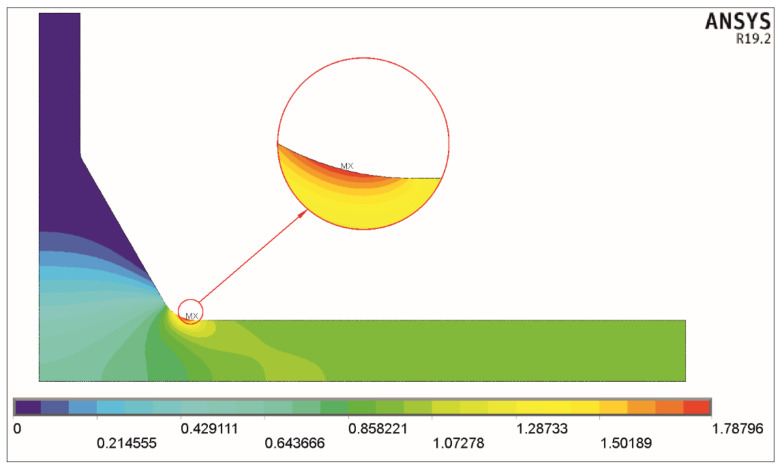
Distribution of the first principal stress *σ*_1_. Tensile load *σ_t_* = 1 MPa, *θ* = 60°, *X* = 0.3, *Y* = 0.4, and *T*/*a* = 1.

**Figure 5:**
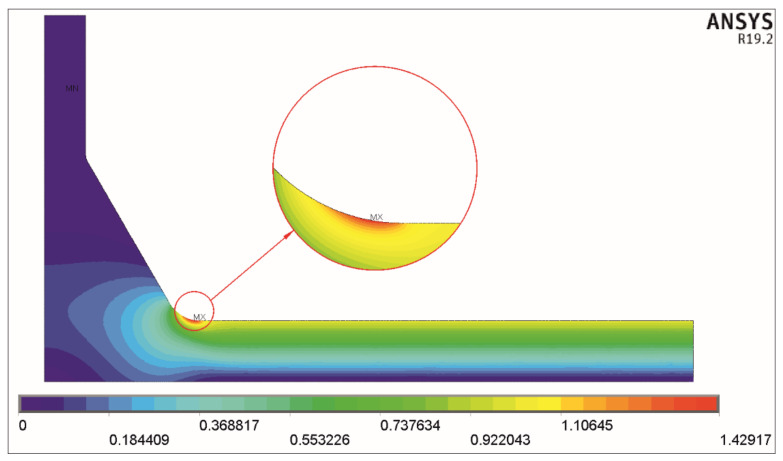
Distribution of the first principal stress *σ*_1_. Bending load *σ_b_* = 1 MPa, *θ* = 60°, *X* = 0.3, *Y* = 0.4, and *T*/*a* = 1.

Two examples of the solution obtained for tension and bending for the nominal stress *σ_t_* = 1 MPa and *σ_b_* = 1 MPa, respectively, are shown in Figure 4 and Figure 5.

In the two cases presented here, the maximum principal stress *σ*_1max_ for axial load equals 1.78796 MPa, and for bending load, it equals 1.42917. These values are directly seen on both scales below the corresponding pictures and represent the stress concentration factors *K_tt_* and *K_tb_*.

### 3.2. Shearing Load

As described in refs. [34,35], an anti-plane state of deformation may be treated as a boundary value problem governed by Laplace’s equation, represented in Cartesian coordinates by Equation (8):

(8) $$\frac{\partial^{2}\Psi }{\partial x^{2}}+\frac{\partial^{2}\Psi }{\partial y^{2}}=0\;$$

The fact that the same relationship is also valid for in-plane steady-state heat conduction problems leads to the conclusion that thermal analogy may be applied to derive stress concentration factors for an anti-plane shear. This approach has been successfully applied in solving anti-plane problems of shearing stress fields in butt and T-joints [34,35].

**Figure 6:**
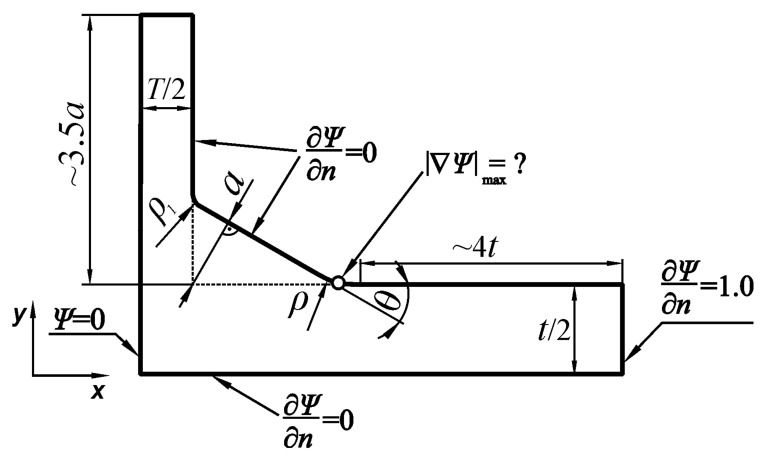
Boundary conditions used in the FEM model for shearing load.

In the present analysis, the ANSYS19 Multiphysics program with a thermal module and PLANE55 finite element was used. A PLANE55 finite element is defined by four nodes with a single degree of freedom corresponding to the temperature at each node. The finite element mesh of the modeled joint was the same as in the previous cases for tensile and bending loads. The shape of the modeled body and mixed boundary conditions are shown in Figure 6.

It was convenient to apply nominal uniform heat flux *q*_nom_ = 1 W/m^2^ along the right end of the body and zero temperature to the left end. Since all remaining faces of the joint are free from external shearing loads, they have to be insulated in the thermal model.

The shear stress components, *τ_xz_* and *τ_yz_*, given by Equation (9):

(9) $$\tau_{xz}=G\frac{\partial W}{\partial x};\;\tau_{yz}=G\frac{\partial W}{\partial y}$$

and related to the partial derivatives of the potential function *W*(*x*,*y*) in particular directions are proportional to the corresponding heat flux components, *q*_x_ and *q*_y_, represented by Equation (10):

(10) $$q_{x}=-k\frac{\partial T_{temp}}{\partial x};\;q_{y}=-k\frac{\partial T_{temp}}{\partial y}.$$

After solving the particular boundary-value problem using the finite element method, numerical SCF values were calculated as a ratio of the maximum magnitude of the temperature gradient (or the maximum heat flux *q*_max_) at the weld toe zone to the magnitude of the nominal temperature gradient (or the nominal heat flux *q*_nom_).

**Figure 7:**
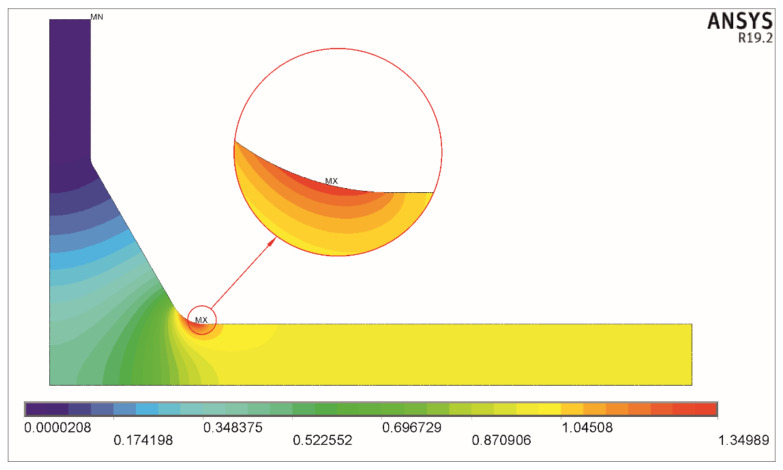
Distribution of the shearing stress *τ.* Shearing load *τ_s_* = 1 MPa, *θ* = 60°, *X* = 0.3, *Y* = 0.4, and *T*/*a* = 1.

One example of a steady-state heat conduction solution is shown in Figure 7. Magnitudes of the flux *q* obtained at each point of the body correspond to the shear stresses *τ.* The maximum *q*_max_ value equals 1.34989 and is shown on the scale below the picture. This value directly represents the *K_ts_* for this particular shape of the joint.

### 3.3. Numerical SCF Results

In subsequent models of cruciform joints, particular values of *X*, *Y*, and *θ* were systematically changed, while the relative attachment thickness *T*/*a* was constant and equal to one. Some examples of such a set of SCF results for a cruciform joint for *θ* = 30°, 40°, 50° and 60° are presented in Table A1, Table A2, Table A3, Table A4, Table A5, Table A6, Table A7, Table A8, Table A9, Table A10, Table A11 and Table A12 given in Appendix B.

A similar 12 sets of SCF data were obtained for other weld angles *θ* in the range of 30–60° with a step of 2.5°. In this way, several thousand SCF numerical solutions were obtained for each loading mode.

## 4. SCF Approximation Formulas

### 4.1. Representation of P Functions

**Figure 8:**
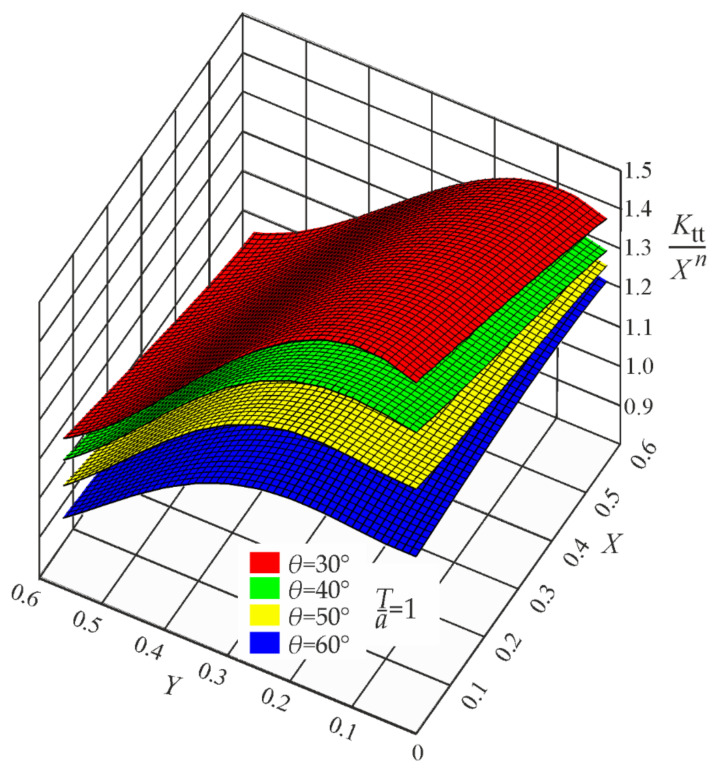
Values of the function $P_{t}=K_{tt}/X^{n}$ for *Z*_0_ = 1, while *θ* = 30°, 40°, 50°, and 60°. Tensile load.

**Figure 9:**
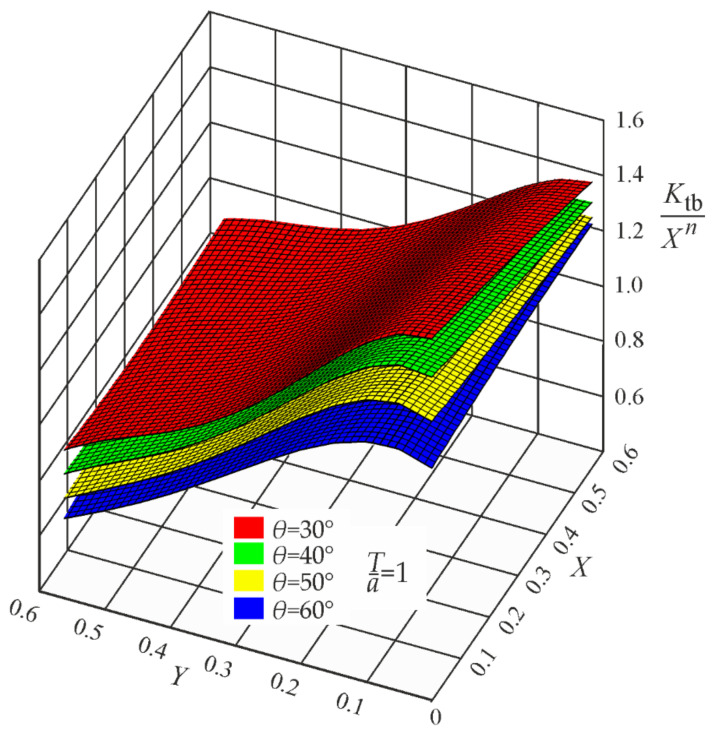
Values of the function $P_{b}=K_{tb}/X^{n}$ for *Z*_0_ = 1, while *θ* = 30°, 40°, 50°, and 60°. Bending load.

**Figure 10:**
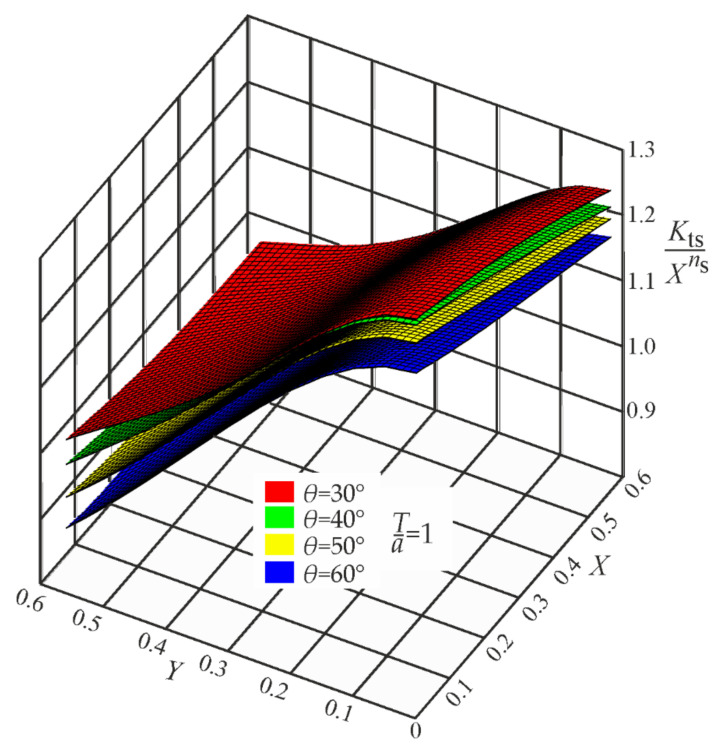
Values of the function $P_{s}=K_{ts}/X^{n_{s}}$ for *Z*_0_ = 1, while *θ* = 30°, 40°, 50°, and 60°. Shearing load.

It is well known that the SCF value becomes infinite when the notch root radius approaches zero. In such a case, any SCF approximating function must be singular, where the $X^{n}$ term is responsible for this singular behavior. After normalizing each *K_t_* value with respect to the properly chosen $X^{n}$ term, three regular functions, *P_t_*, *P_b_*, and *P_s_*, may be obtained. Their mathematical description by polynomials is shown in the brackets of Equations (A1)–(A3) given in Appendix A. Graphical representations of these functions, corresponding to the weld angle *θ* = 30°, 40°, 50° and 60° for three loading modes, are shown in Figure 8, Figure 9 and Figure 10.

### 4.2. Validation of the P Functions

The accuracy of the approximating *P* functions, represented by polynomials shown in Appendix A, was verified for all loading modes, while the weld angle *θ* varied in the range of 30°–60°. Particular SCF values (*) calculated from Equation (11)

(11) $$K_{t}=X^{n}P\left(X,Y,\theta ,Z_{0}\right),$$

were compared to their numerical equivalencies obtained using the finite element method. Equation (11) is represented by the parametric formulas (A1)–(A3), when *Z* = *Z*_0_ = 1 and *κ* = 1. Three examples of such comparisons are presented in Table 1, Table 2 and Table 3 for tension, bending, and shear.

Similar comparisons were made for various weld angles *θ* in the range of 30°–60°. The maximum percentage error of the approximation was lower than 2.0%. Some examples of graphical representations of SCFs vs. *X* for different values of *θ* and *Y* are shown in Appendix C.

### 4.3. Determination of the Correction Functions κ

The influence of the relative attachment plate thickness *Z* = *T*/*a* on the SCF was determined by performing additional FEM modeling. Several thousand new cases of a cruciform joint were modeled and solved for each loading mode. Particular values of *T*/*a* were changed in the range of 1 ≤ *T*/*a* ≤ 4. Additional correction function *κ* was introduced to express these effects.

The general mathematical form of the correction functions *κ*, given by Equation (12),

(12) $$\kappa \left(X,Y,Z,\theta ,Z_{0}\right)=1+\left(\sqrt{Z}-\sqrt{Z_{0}}\right)\left[1-\left(B_{1}+B_{2}Y^{2}\right)X^{m}\right]Exp\left[-{\left(B_{3}Y\right)}^{p}-B_{4}\right]$$

is the same as in Reference [35] developed by the authors for a welded T-joint. The particular values of the coefficients *B*_1_–*B*_4_ and the exponents *m* and *p* were derived for each loading mode using the least-squares method. The best fits of the functions *κ_t_*, *κ_b_* and *κ_s_* to the numerical FEM SCF results were obtained as follows: *m* = 1 for tensile and bending load and *m* = 2 for shearing load. The particular *p* values were equal to 2.7, 2.0, and 2.0 for tensile, bending, and shearing loads, respectively.

**Figure 11:**
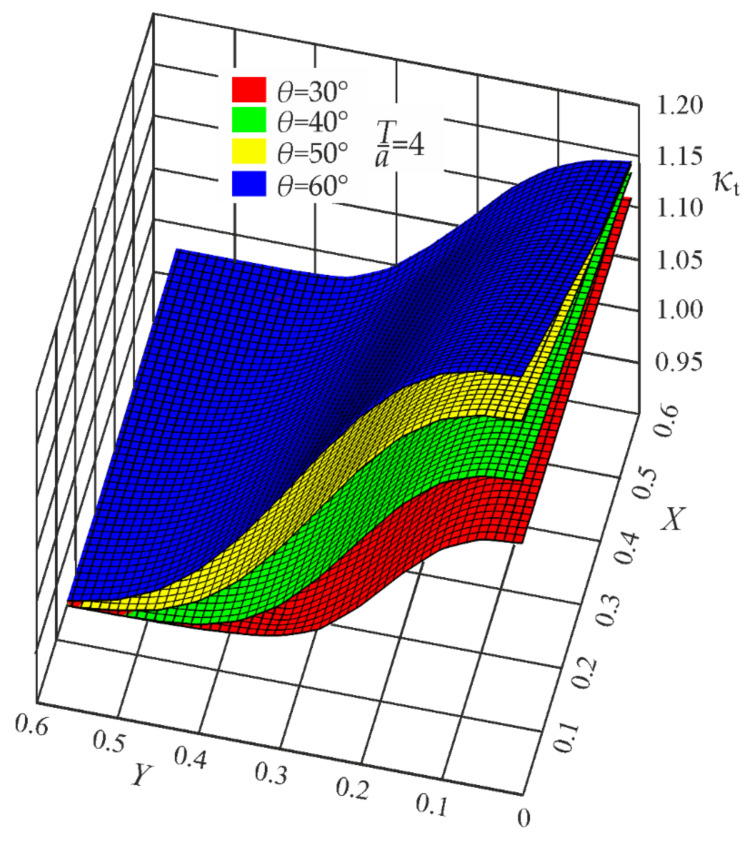
Correction functions *κ_t_* for *θ* = 30°, 40°, 50°, and 60°. Tensile load.

**Figure 12:**
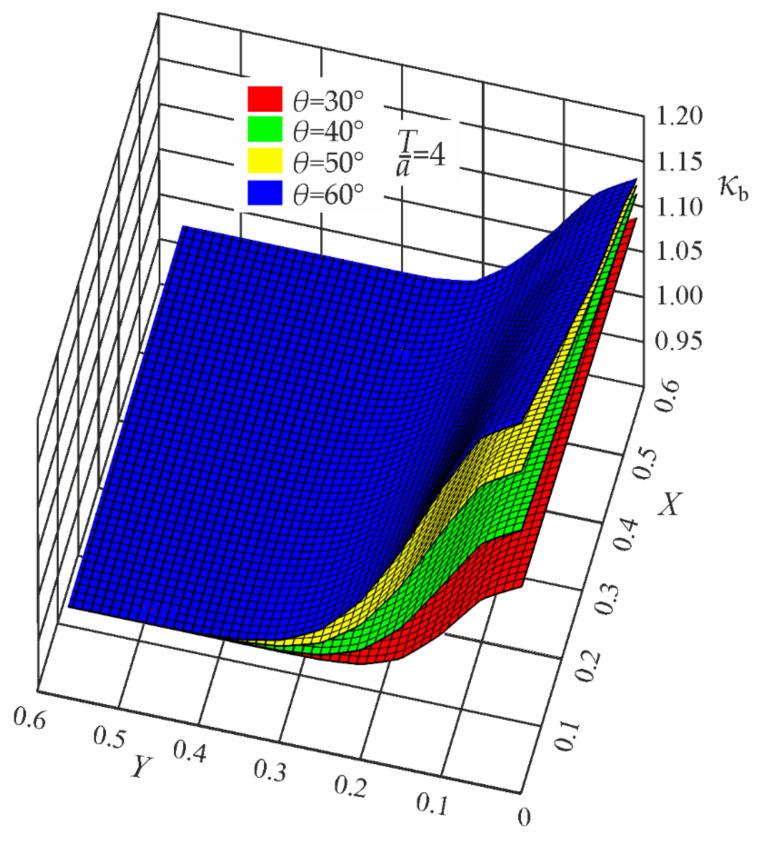
Correction functions *κ_b_* for *θ* = 30°, 40°, 50°, and 60°. Bending load.

**Figure 13:**
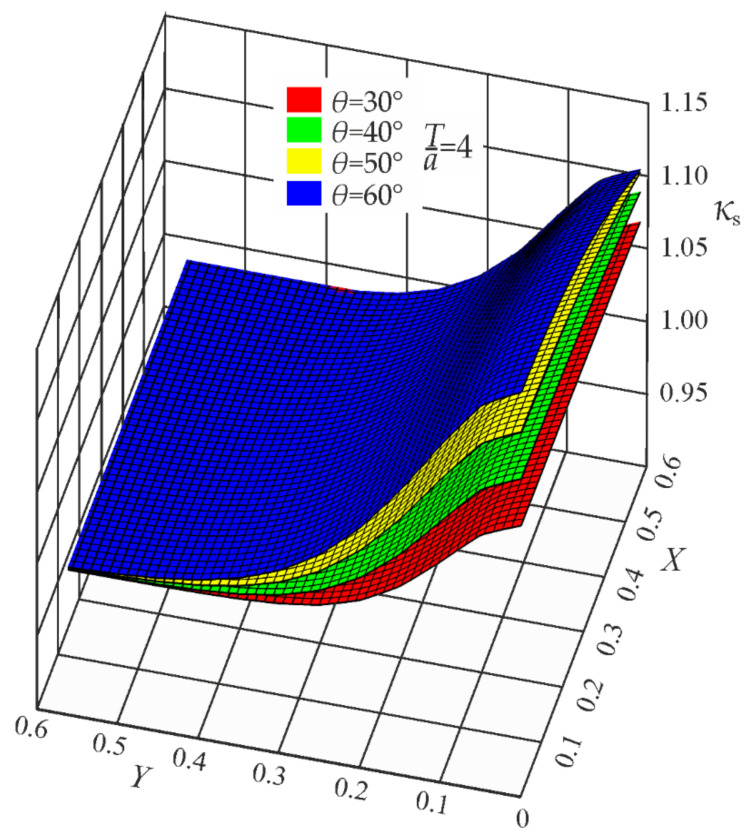
Correction functions *κ_s_* for *θ* = 30°, 40°, 50°, and 60°. Shearing load.

Since in the present case the coefficients *B*_i_ depend on the weld angle *θ*, additional functions *B*_i_(*θ*) had to be developed. Parametric formulas of the correction functions *κ_t_*, *κ_b_*, and *κ_s_* are given in Appendix A. Some geometrical representations of the correction functions *κ_t_*, *κ_b_*, and *κ_s_*, for *T*/*a* = 4, are depicted in Figure 11, Figure 12 and Figure 13.

### 4.4. Validation of SCF Parametric Equations

The accuracy of the SCF parametric equations, given in Appendix A, was verified for all loading modes, while the weld angle *θ* varied in the range of 30°–60°. Examples of such a comparison are shown in Table 4, Table 5 and Table 6 for the angle *θ* equal to 30°, 45°, and 60°.

The same validation was carried out for weld angles *θ* changing with a step of 2.5°. The maximum error, calculated with respect to the SCF values obtained using the FEM was much lower than 2.5%.

## 5. Conclusions

Several thousand numerical FEM models of cruciform connections subjected to tensile, bending, and shearing loads were performed. The particular values of SCFs obtained from numerical solutions enabled the derivation of three approximating parametric formulas, covering the dimensions of welded cruciform connections used in real structures. Five characteristic geometrical parameters: *t*, *T*, *a*, *ρ*, and *θ* describing the shape of the joint were considered as independent variables. The accuracy of the formulas is higher than 97.5% and covers very wide ranges of application: 0 < *ρ*/*a* ≤ 1.3, 0 < *a*/*t* ≤ 1.3, 1 ≤ *T*/*a* ≤ 4, and 30° ≤ *θ*≤60°, including two limiting cases: for *ρ*→0 and when *t*→∞. In this way, all possible values of all geometrical parameters used in real cruciform joints are satisfied.

It is also important to note that the approximating equations presented herein agree with the results and formulas published in [32] and are valid for *θ* = 45°.

The use of such SCFs’ solutions is recommended in EN 1993-1-9: 2005 [21] (Design of steel structures, Part 1–9: Fatigue, Sections 5 and 6. See Table A1 case 4 in addition). In such cases, the recommendations in Section 6.3 of ref. [21] state that… “SCF values may be taken from handbooks or from appropriate FE calculations”.

The proposed formulas can be easily applied in computer-aided assessment in the fatigue design of cruciform joints, particularly in:

- Comparative studies of the stress concentration in cruciform joints of different dimensions;
- Hot spot approaches;
- The weight function method used for cracks initiated at the weld toe;
- Dealing with the possible mechanical improvements in the weld toe region.

It is important to note that the accuracy of the parametric formulas depends on the similarity of the real joint shape to the one assumed in the present models used in the numerical analysis. Wang et al. [17] have shown that the improper identification or interpretation of geometrical parameters and the shape of the joint may lead to significant errors in SCF estimation. The same may occur when the geometrical parameters’ values lay beyond the specified range of validity.

Future work should be focused on local effects appearing in the critical zone where damage processes occur. It is usually assumed that small cracks initiate due to very high local stress ranges and there are at least two possible classes of methods to attack this problem. First, methods based on fracture mechanics may be used. Theoretical, regular, or singular stress fields (when *ρ* tends to zero) around the apex are averaged in small volumes or areas that are material constants. Second, there are methods of analyzing fatigue crack growth using weight functions, assuming that the fatigue life depends on the propagating crack. However, the aim of the present paper did not cover such problems.

## Figures and Tables

**Table 1:** *K_tt_* values obtained using the FEM and calculated from Equation (A1) (*). Tensile load, *θ* = 30°, and *T*/*a* = 1.

*θ* = 30°	*X*						
*Y*	0.050	0.100	0.200	0.300	0.400	0.500	0.562
0.050	3.530→3.563 *	2.933→2.960 *	2.404→2.424 *	2.112→2.128 *	1.904→1.920 *	1.739→1.755 *	1.649→1.667 *
0.100	3.545→3.570 *	2.946→2.966 *	2.414→2.429 *	2.120→2.132 *	1.912→1.922 *	1.745→1.756 *	1.654→1.667 *
0.200	3.444→3.453 *	2.862→2.869 *	2.343→2.348 *	2.056→2.059 *	1.850→1.854 *	1.685→1.690 *	1.593→1.600 *
0.300	3.166→3.202 *	2.630→2.660 *	2.153→2.176 *	1.887→1.907 *	1.697→1.715 *	1.543→1.561 *	1.458→1.477 *
0.400	2.859→2.882 *	2.375→2.393 *	1.944→1.958 *	1.705→1.716 *	1.534→1.544 *	1.398→1.408 *	1.324→1.335 *
0.500	2.582→2.576 *	2.145→2.139 *	1.757→1.750 *	1.543→1.536 *	1.393→1.386 *	1.278→1.272 *	1.218→1.215 *
0.562	2.423→2.437 *	2.014→2.023 *	1.652→1.656 *	1.454→1.455 *	1.318→1.317 *	1.217→1.216 *	1.168→1.168 *

**Table 2:** *K_tb_* values obtained using the FEM and calculated from Equation (A2) (*). Bending load, *θ* = 60°, and *T*/*a* = 1.

*θ* = 60°	*X*						
*Y*	0.050	0.100	0.200	0.300	0.400	0.500	0.562
0.050	4.047→4.043 *	3.107→3.105 *	2.387→2.387 *	2.044→2.045 *	1.827→1.828 *	1.671→1.671 *	1.592→1.591 *
0.100	4.030→4.012 *	3.085→3.073 *	2.360→2.351 *	2.011→2.005 *	1.790→1.785 *	1.629→1.624 *	1.546→1.542 *
0.200	3.721→3.701 *	2.838→2.824 *	2.156→2.148 *	1.827→1.823 *	1.617→1.616 *	1.464→1.465 *	1.385→1.389 *
0.300	3.244→3.245 *	2.472→2.477 *	1.886→1.887 *	1.606→1.607 *	1.432→1.432 *	1.308→1.308 *	1.250→1.247 *
0.400	2.793→2.797 *	2.143→2.147 *	1.654→1.656 *	1.429→1.431 *	1.297→1.296 *	1.207→1.205 *	1.164→1.161 *
0.500	2.418→2.423 *	1.874→1.880 *	1.477→1.483 *	1.304→1.311 *	1.205→1.212 *	1.141→1.148 *	1.111→1.118 *
0.562	2.212→2.223 *	1.730→1.739 *	1.387→1.393 *	1.242→1.245 *	1.161→1.162 *	1.109→1.108 *	1.085→1.082 *

**Table 3:** *K_ts_* values obtained using the FEM and calculated from Equation (A3) (*). Shearing load, *θ* = 45°, and *T*/*a* = 1.

*θ* = 45°	*X*						
*Y*	0.050	0.100	0.200	0.300	0.400	0.500	0.562
0.0256	2.497→2.500 *	2.152→2.155 *	1.835→1.836 *	1.655→1.656 *	1.527→1.528 *	1.425→1.428 *	1.370→1.373 *
0.0526	2.487→2.487 *	2.143→2.144 *	1.827→1.827 *	1.648→1.647 *	1.520→1.520 *	1.419→1.420 *	1.364→1.366 *
0.1111	2.440→2.436 *	2.102→2.099 *	1.792→1.789 *	1.616→1.613 *	1.491→1.489 *	1.391→1.391 *	1.337→1.337 *
0.1765	2.353→2.351 *	2.028→2.026 *	1.729→1.727 *	1.559→1.558 *	1.438→1.439 *	1.343→1.345 *	1.291→1.293 *
0.2500	2.234→2.236 *	1.925→1.927 *	1.642→1.643 *	1.483→1.484 *	1.369→1.372 *	1.281→1.285 *	1.233→1.238 *
0.3333	2.095→2.098 *	1.807→1.808 *	1.543→1.543 *	1.396→1.397 *	1.294→1.296 *	1.216→1.219 *	1.175→1.177 *
0.3908	2.003→2.004 *	1.728→1.728 *	1.478→1.477 *	1.341→1.340 *	1.247→1.248 *	1.177→1.178 *	1.142→1.140 *

**Table 4:** Comparison of SCF values obtained from Equations (A1)–(A3) (*) to the corresponding FEM results for *θ* = 30°.

*θ* = 30°		*K_tt_*				*K_tb_*				*K_ts_*			
*ρ*/*a*	*t*/*a*	*T*/*a* = 1	*T*/*a* = 2	*T*/*a* = 3	*T*/*a* = 4	*T*/*a* = 1	*T*/*a* = 2	*T*/*a* = 3	*T*/*a* = 4	*T*/*a* = 1	*T*/*a* = 2	*T*/*a* = 3	*T*/*a* = 4
0.05	10	3.599 3.618 *	3.769 3.807 *	3.918 3.951 *	4.047 4.074 *	3.455 3.452 *	3.553 3.545 *	3.624 3.616 *	3.676 3.677 *	2.113 2.114 *	2.158 2.163 *	2.196 2.201 *	2.229 2.233 *
	7	3.598 3.602 *	3.753 3.761 *	3.878 3.884 *	3.977 3.987 *	3.318 3.330 *	3.375 3.379 *	3.410 3.417 *	3.430 3.448 *	2.077 2.078 *	2.111 2.113 *	2.137 2.139 *	2.158 2.162 *
	4	3.495 3.497 *	3.580 3.573 *	3.633 3.631 *	3.668 3.679 *	2.986 3.013 *	2.996 3.019 *	3.000 3.023 *	3.001 3.027 *	1.983 1.985 *	1.997 1.997 *	2.006 2.007 *	2.014 2.015 *
0.25	10	2.415 2.430 *	2.526 2.553 *	2.623 2.648 *	2.707 2.727 *	2.318 2.318 *	2.382 2.377 *	2.428 2.423 *	2.461 2.461 *	1.680 1.682 *	1.715 1.721 *	1.746 1.751 *	1.771 1.776 *
	7	2.412 2.419 *	2.513 2.522 *	2.594 2.601 *	2.659 2.668 *	2.227 2.237 *	2.263 2.267 *	2.285 2.290 *	2.299 2.310 *	1.651 1.654 *	1.678 1.681 *	1.698 1.702 *	1.715 1.719 *
	4	2.343 2.348 *	2.399 2.395 *	2.434 2.431 *	2.457 2.462 *	2.008 2.026 *	2.014 2.029 *	2.016 2.032 *	2.017 2.034 *	1.576 1.579 *	1.588 1.589 *	1.595 1.597 *	1.600 1.603 *
0.5	10	2.045 2.057 *	2.137 2.158 *	2.218 2.236 *	2.289 2.301 *	1.961 1.959 *	2.012 2.006 *	2.051 2.043 *	2.078 2.073 *	1.523 1.526 *	1.555 1.560 *	1.582 1.587 *	1.606 1.609 *
	7	2.043 2.047 *	2.126 2.131 *	2.193 2.195 *	2.248 2.249 *	1.883 1.890 *	1.912 1.914 *	1.930 1.932 *	1.941 1.948 *	1.497 1.500 *	1.521 1.524 *	1.539 1.543 *	1.554 1.558 *
	4	1.983 1.984 *	2.027 2.021 *	2.055 2.050 *	2.074 2.074 *	1.689 1.715 *	1.702 1.717 *	1.704 1.719 *	1.704 1.721 *	1.430 1.433 *	1.439 1.441 *	1.446 1.448 *	1.451 1.453 *
1	10	1.746 1.758 *	1.819 1.842 *	1.883 1.906 *	1.940 1.960 *	1.670 1.666 *	1.710 1.704 *	1.739 1.733 *	1.760 1.758 *	1.385 1.384 *	1.412 1.415 *	1.436 1.438 *	1.456 1.458 *
	7	1.743 1.747 *	1.807 1.816 *	1.860 1.868 *	1.903 1.913 *	1.604 1.608 *	1.625 1.627 *	1.638 1.641 *	1.647 1.653 *	1.361 1.361 *	1.381 1.382 *	1.397 1.398 *	1.410 1.412 *
	4	1.685 1.690 *	1.718 1.718 *	1.739 1.741 *	1.753 1.759 *	1.454 1.465 *	1.454 1.467 *	1.454 1.468 *	1.455 1.469 *	1.301 1.301 *	1.309 1.308 *	1.314 1.314 *	1.318 1.318 *

**Table 5:** Comparison of SCF values obtained from Equations (A1)–(A3) (*) to the corresponding FEM results for *θ* = 45°.

*θ* = 45°		*K_tt_*				*K_tb_*				*K_ts_*			
*ρ*/*a*	*t*/*a*	*T*/*a* = 1	*T*/*a* = 2	*T*/*a* = 3	*T*/*a* = 4	*T*/*a* = 1	*T*/*a* = 2	*T*/*a* = 3	*T*/*a* = 4	*T*/*a* = 1	*T*/*a* = 2	*T*/*a* = 3	*T*/*a* = 4
0.05	10	4.036 4.034 *	4.361 4.394 *	4.645 4.671 *	4.893 4.904 *	3.996 3.982 *	4.249 4.223 *	4.438 4.408 *	4.579 4.564 *	2.485 2.482 *	2.585 2.585 *	2.666 2.663 *	2.732 2.730 *
	7	4.051 4.042 *	4.372 4.378 *	4.640 4.635 *	4.860 4.853 *	3.897 3.881 *	4.084 4.045 *	4.206 4.171 *	4.284 4.278 *	2.449 2.445 *	2.530 2.525 *	2.592 2.586 *	2.639 2.637 *
	4	4.037 4.011 *	4.295 4.253 *	4.478 4.439 *	4.609 4.596 *	3.562 3.562 *	3.630 3.608 *	3.661 3.643 *	3.677 3.673 *	2.340 2.340 *	2.385 2.379 *	2.413 2.408 *	2.433 2.434 *
0.25	10	2.439 2.445 *	2.620 2.642 *	2.778 2.794 *	2.918 2.921 *	2.403 2.400 *	2.541 2.527 *	2.646 2.625 *	2.724 2.708 *	1.807 1.804 *	1.878 1.877 *	1.935 1.932 *	1.982 1.979 *
	7	2.446 2.447 *	2.624 2.629 *	2.773 2.769 *	2.898 2.886 *	2.341 2.336 *	2.441 2.420 *	2.507 2.485 *	2.550 2.540 *	1.780 1.777 *	1.831 1.833 *	1.882 1.876 *	1.918 1.913 *
	4	2.430 2.422 *	2.568 2.547 *	2.666 2.644 *	2.737 2.725 *	2.140 2.142 *	2.174 2.164 *	2.190 2.180 *	2.197 2.194 *	1.702 1.701 *	1.733 1.728 *	1.753 1.749 *	1.767 1.766 *
0.5	10	2.009 2.015 *	2.143 2.163 *	2.263 2.276 *	2.369 2.372 *	1.969 1.966 *	2.068 2.058 *	2.143 2.128 *	2.201 2.188 *	1.584 1.581 *	1.642 1.642 *	1.691 1.688 *	1.730 1.727 *
	7	2.015 2.015 *	2.146 2.150 *	2.257 2.253 *	2.351 2.339 *	1.913 1.911 *	1.983 1.970 *	2.029 2.015 *	2.059 2.053 *	1.560 1.558 *	1.608 1.604 *	1.644 1.639 *	1.672 1.669 *
	4	1.993 1.987 *	2.091 2.076 *	2.161 2.144 *	2.211 2.201 *	1.746 1.753 *	1.767 1.765 *	1.776 1.775 *	1.780 1.784 *	1.492 1.492 *	1.517 1.514 *	1.533 1.530 *	1.544 1.544 *
1	10	1.698 1.703 *	1.793 1.812 *	1.878 1.895 *	1.955 1.966 *	1.651 1.648 *	1.716 1.712 *	1.766 1.761 *	1.803 1.802 *	1.403 1.402 *	1.448 1.450 *	1.486 1.486 *	1.517 1.517 *
	7	1.701 1.699 *	1.791 1.796 *	1.868 1.870 *	1.933 1.933 *	1.600 1.599 *	1.642 1.638 *	1.670 1.667 *	1.688 1.692 *	1.382 1.382 *	1.417 1.417 *	1.445 1.445 *	1.467 1.467 *
	4	1.672 1.665 *	1.732 1.723 *	1.775 1.768 *	1.806 1.806 *	1.462 1.469 *	1.471 1.475 *	1.474 1.480 *	1.476 1.484 *	1.323 1.326 *	1.340 1.341 *	1.351 1.353 *	1.359 1.363 *

**Table 6:** Comparison of SCF values obtained from Equations (A1)–(A3) (*) to the corresponding FEM results for *θ* = 60°.

*θ* = 60°		*K_tt_*				*K_tb_*				*K_ts_*			
*ρ*/*a*	*t*/*a*	*T*/*a* = 1	*T*/*a* = 2	*T*/*a* = 3	*T*/*a* = 4	*T*/*a* = 1	*T*/*a* = 2	*T*/*a* = 3	*T*/*a* = 4	*T*/*a* = 1	*T*/*a* = 2	*T*/*a* = 3	*T*/*a* = 4
0.05	10	4.087 4.061 *	4.514 4.535 *	4.892 4.899 *	5.228 5.206 *	4.125 4.102 *	4.507 4.474 *	4.808 4.760 *	5.041 5.000 *	2.773 2.763 *	2.935 2.921 *	3.061 3.043 *	3.163 3.145 *
	7	4.107 4.076 *	4.540 4.531 *	4.911 4.881 *	5.233 5.175 *	4.066 4.035 *	4.382 4.314 *	4.600 4.529 *	4.752 4.709 *	2.736 2.725 *	2.874 2.853 *	2.976 2.951 *	3.051 3.035 *
	4	4.127 4.081 *	4.517 4.457 *	4.816 4.746 *	5.045 4.990 *	3.795 3.772 *	3.947 3.879 *	4.026 3.961 *	4.070 4.030 *	2.618 2.612 *	2.703 2.682 *	2.756 2.736 *	2.791 2.782 *
0.25	10	2.378 2.373 *	2.585 2.608 *	2.769 2.789 *	2.935 2.941 *	2.368 2.362 *	2.546 2.537 *	2.686 2.671 *	2.797 2.784 *	1.876 1.870 *	1.978 1.972 *	2.059 2.050 *	2.124 2.116 *
	7	2.386 2.378 *	2.593 2.601 *	2.773 2.772 *	2.928 2.916 *	2.324 2.313 *	2.465 2.440 *	2.564 2.537 *	2.632 2.619 *	1.850 1.844 *	1.937 1.926 *	2.002 1.989 *	2.050 2.042 *
	4	2.383 2.368 *	2.563 2.544 *	2.699 2.678 *	2.802 2.792 *	2.156 2.148 *	2.216 2.191 *	2.246 2.224 *	2.262 2.252 *	1.771 1.768 *	1.823 1.812 *	1.855 1.846 *	1.877 1.875 *
0.5	10	1.963 1.965 *	2.109 2.129 *	2.240 2.255 *	2.359 2.361 *	1.937 1.934 *	2.055 2.048 *	2.148 2.137 *	2.221 2.211 *	1.609 1.602 *	1.687 1.681 *	1.750 1.741 *	1.801 1.793 *
	7	1.969 1.967 *	2.114 2.120 *	2.239 2.237 *	2.347 2.336 *	1.893 1.887 *	1.981 1.967 *	2.043 2.028 *	2.085 2.080 *	1.586 1.579 *	1.652 1.642 *	1.701 1.690 *	1.738 1.730 *
	4	1.958 1.948 *	2.075 2.062 *	2.162 2.149 *	2.228 2.223 *	1.744 1.745 *	1.766 1.768 *	1.791 1.785 *	1.799 1.800 *	1.518 1.515 *	1.557 1.548 *	1.579 1.572 *	1.595 1.593 *
1	10	1.680 1.678 *	1.778 1.786 *	1.867 1.869 *	1.947 1.939 *	1.639 1.635 *	1.709 1.703 *	1.764 1.754 *	1.807 1.798 *	1.410 1.401 *	1.464 1.455 *	1.508 1.497 *	1.545 1.533 *
	7	1.683 1.676 *	1.777 1.773 *	1.859 1.848 *	1.928 1.911 *	1.593 1.590 *	1.640 1.632 *	1.672 1.664 *	1.694 1.692 *	1.390 1.381 *	1.433 1.422 *	1.466 1.454 *	1.492 1.481 *
	4	1.657 1.645 *	1.725 1.709 *	1.774 1.758 *	1.809 1.799 *	1.464 1.465 *	1.471 1.471 *	1.476 1.476 *	1.478 1.480 *	1.330 1.327 *	1.352 1.346 *	1.366 1.360 *	1.376 1.372 *

**Figure A1:**
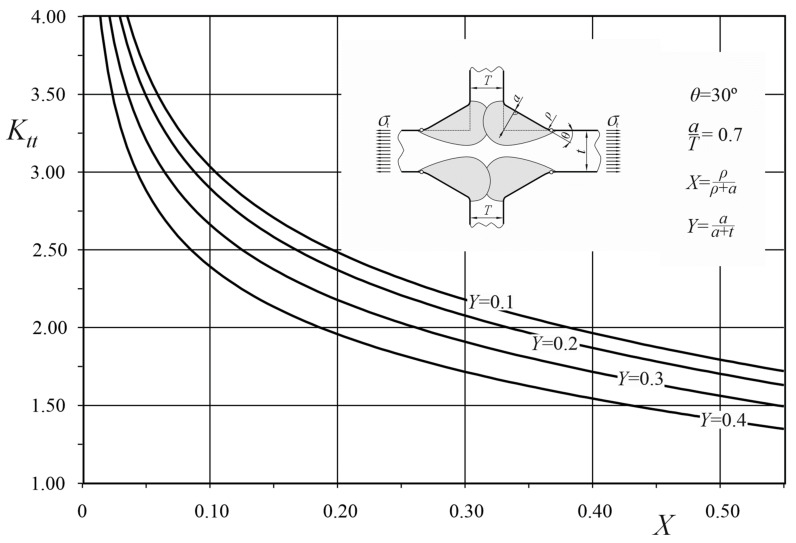
*K_tt_* vs. *X* for *a*/*T* = 0.7 and *θ* = 30°. Tensile load.

**Figure A2:**
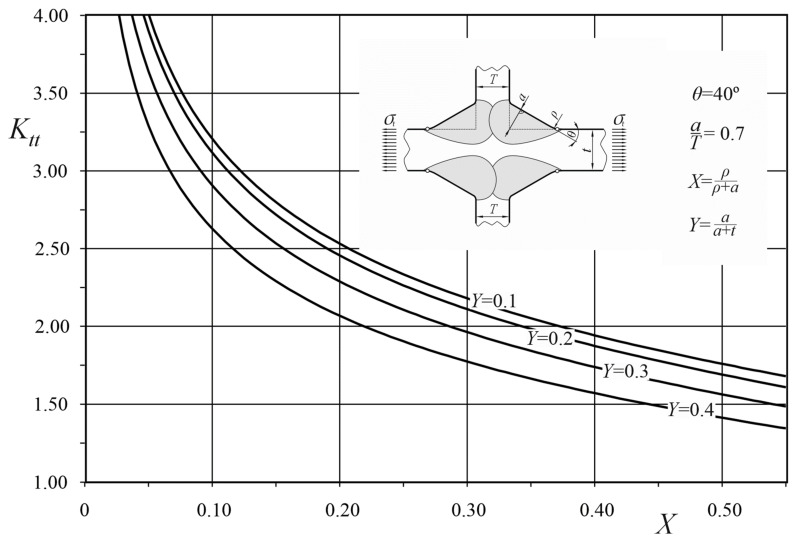
*K_tt_* vs. *X* for *a*/*T* = 0.7 and *θ* = 40°. Tensile load.

**Figure A3:**
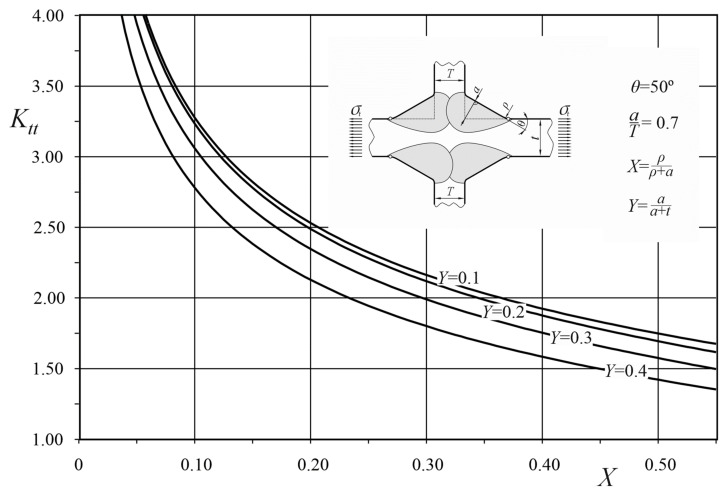
*K_tt_* vs. *X* for *a*/*T* = 0.7 and *θ* = 50°. Tensile load.

**Figure A4:**
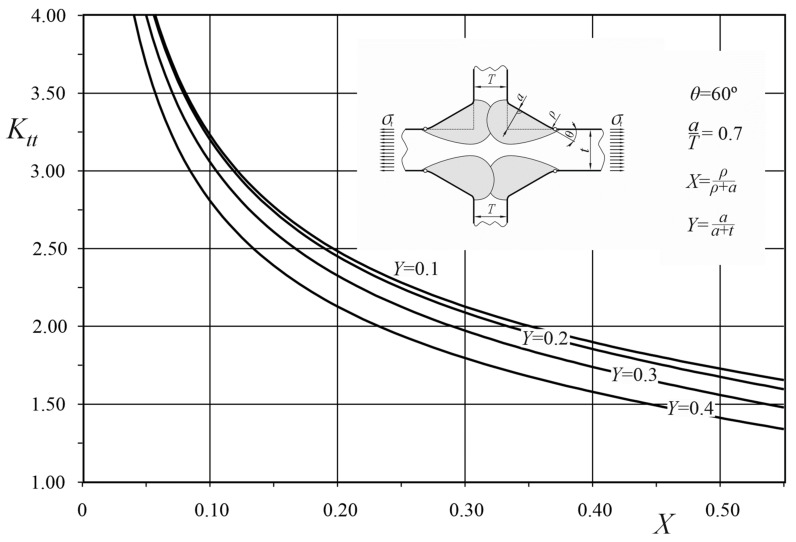
*K_tt_* vs. *X* for *a*/*T* = 0.7 and *θ* = 60°. Tensile load.

**Figure A5:**
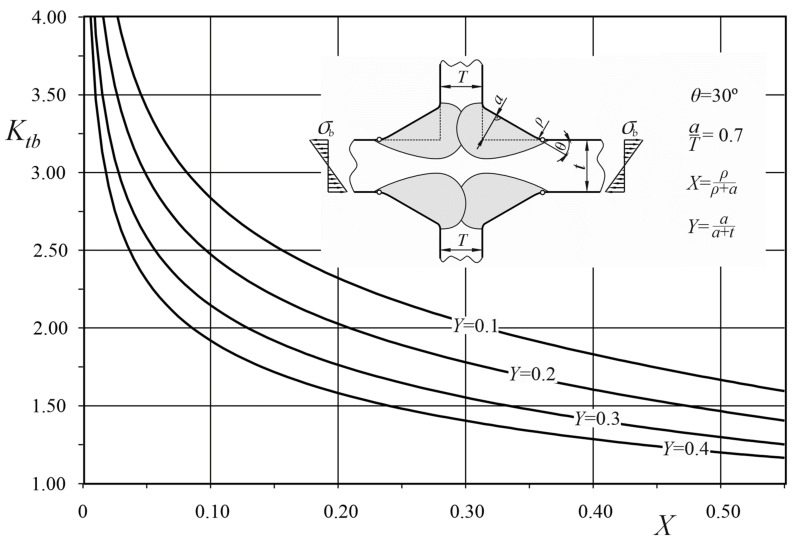
*K_tb_* vs. *X* for *a*/*T* = 0.7 and *θ* = 30°. Bending load.

**Figure A6:**
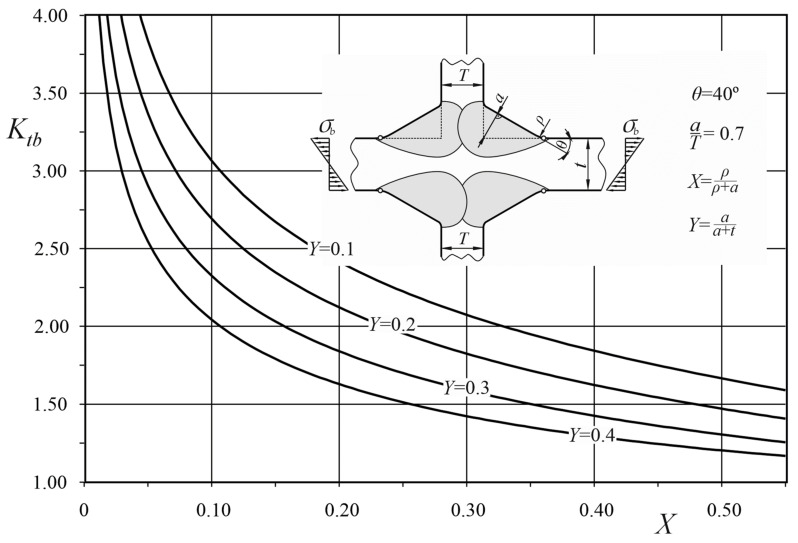
*K_tb_* vs. *X* for *a*/*T* = 0.7 and *θ* = 40°. Bending load.

**Figure A7:**
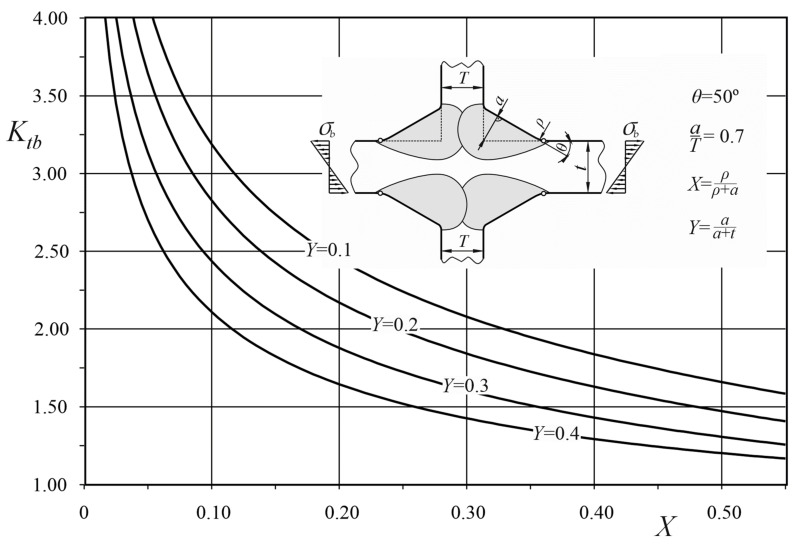
*K_tb_* vs. *X* for *a*/*T* = 0.7 and *θ* = 50°. Bending load.

**Figure A8:**
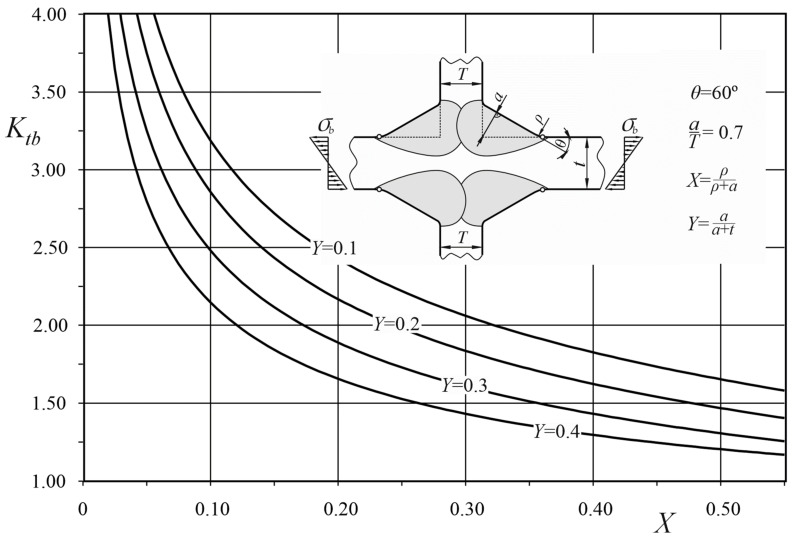
*K_tb_* vs. *X* for *a*/*T* = 0.7 and *θ* = 60°. Bending load.

**Figure A9:**
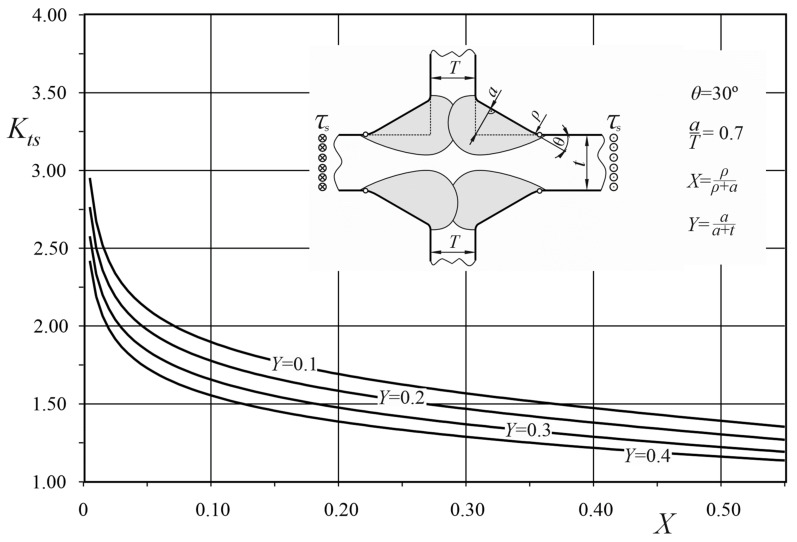
*K_ts_* vs. *X* for *a*/*T* = 0.7 and *θ* = 30°. Shearing load.

**Figure A10:**
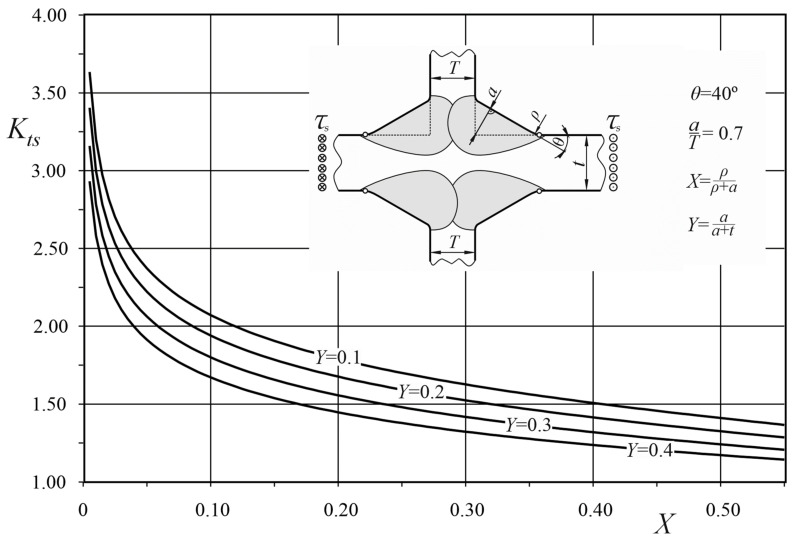
*K_ts_* vs. *X* for *a*/*T* = 0.7 and *θ* = 40°. Shearing load.

**Figure A11:**
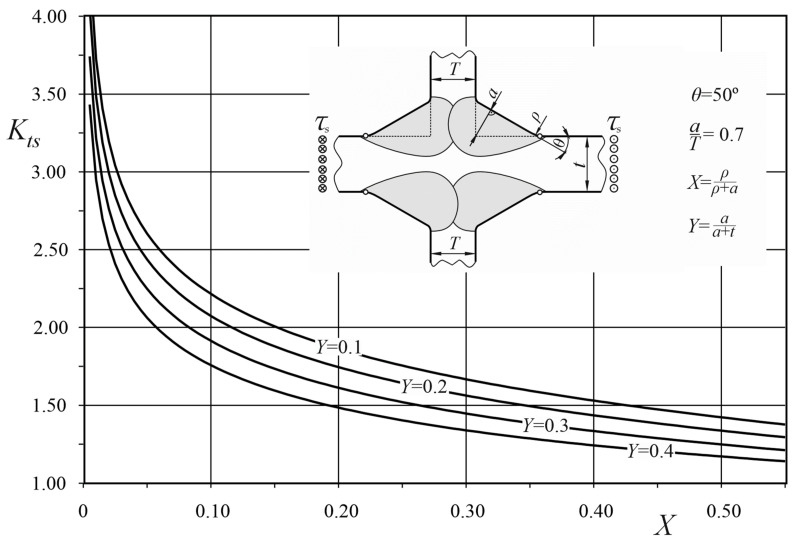
*K_ts_* vs. *X* for *a*/*T* = 0.7 and *θ* = 50°. Shearing load.

**Figure A12:**
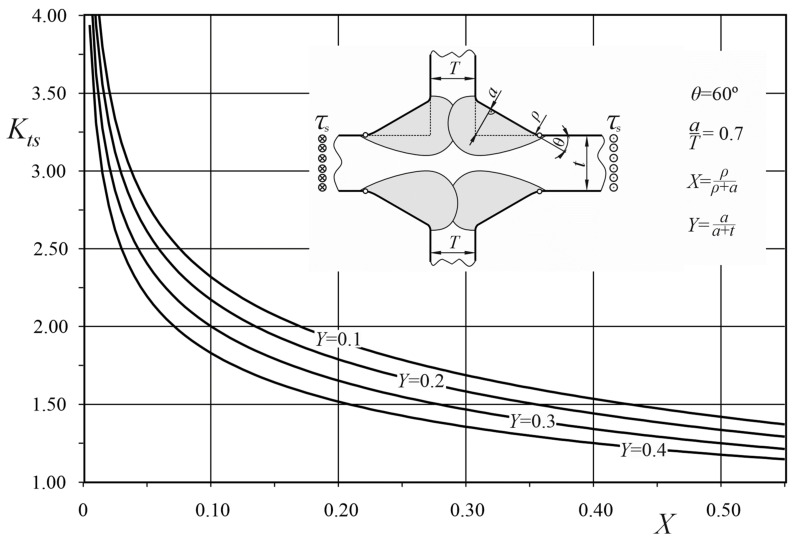
*K_ts_* vs. *X* for *a*/*T* = 0.7 and *θ* = 60°. Shearing load.

## Data Availability

Data are contained within the article.

## Nomenclature

*a*	weld throat thickness
G	shear modulus
*h*, *h_p_*	leg lengths
*k*	thermal conductivity
*K_t_*	stress concentration factor (SCF)
*K_tt_*	stress concentration factor for tensile (axial) load
*K_tb_*	stress concentration factor for bending load
*K_ts_*	stress concentration factor for shearing load
*m*, *p*	exponents dependent on the loading mode
*n*	stress field exponent for a sharp corner for axial and bending load
*n_s_*	stress field exponent for a sharp corner subjected to shearing load
*P*	regular function represented by polynomials
*P_t_*, *P_b_*, *P_s_*	*P* functions corresponding to tensile, bending, and shearing load, respectively
*Q*	magnitude of the heat flux
*q*_max_	magnitude of the maximum heat flux
*q*_nom_	magnitude of the nominal heat flux at the end of the body
*t*	thickness of the main plate
*T*	thickness of the attachment plates
*T*_temp_	temperature
*W*	displacement component corresponding to the anti-plane shear
*X* = *ρ*/(*ρ* + *a*)	normalized toe radius parameter
*Y* = *a*/(*a* + *t*)	normalized weld thickness parameter
*Z* = *T*/*a*	normalized attachment plate thickness parameter
*Z*_0_	reference *T*/*a* value
|∇*Ψ*|	magnitude of the displacement (temperature) gradient
|∇*Ψ*|_max_	magnitude of the maximum displacement (temperature) gradient
|∇*Ψ*|_nom_	magnitude of the nominal displacement (temperature) gradient
*θ*	weld angle
*κ*	correction function for the attachment plate thickness *T*/*a*
*κ_t_*	correction function *κ* for tensile (axial) load
*κ_b_*	correction function *κ* for bending load
*κ_s_*	correction function *κ* for shearing load
*Ψ*(*x*,*y*)	potential function representing temperature or anti-plane displacement
∂*Ψ*/∂*n*	normal derivative at the bounding contour
*ρ*	weld toe radius
*σ*_1_	first principal stress for tensile or bending load
*σ*_1max_	maximum value of the first principal stress
*σ_t_*	nominal tensile (axial) stress
*σ_b_*	nominal bending stress
*τ*_max_	maximum shear stress at the weld toe due to shearing load
*τ*_s_	nominal shear stress

## Appendix A

Approximating SCF formulas for the cruciform connections subjected to tensile, bending, and shearing loads

Independent variables: *ρ*, *a*, *θ*, *t*, *T*;

Normalized quantities: *X* = *ρ*/(*ρ* + *a*), *Y* = *a*/(*a* + *t*); *Z* = *T*/*a*;

Range of validity: 0 < *ρ*/*a* ≤ 1.3; 0 < *a*/*t* ≤ 1.3; 1 ≤ *T*/*a* ≤ 4; 30° ≤ *θ* ≤ 60°.

In all formulas, *θ* is expressed in radians.

Exponent of the singular term for:

axial and bending load

$$n=\frac{-0.63662\theta -0.09330\theta^{2}}{1+0.77635\theta +0.04075\theta^{1.5}-0.00499\theta^{2}+0.13365\theta^{2.5}}$$

shearing load

$$n_{s}=\frac{-\theta }{\theta +\pi }$$

Accuracy: maximum percentage error lower than 2.5% compared to the FEM results.

Tensile (axial) load

(A1) $$K_{tt}=X^{n}\left(A_{0}^{t}+A_{1}^{t}X+A_{2}^{t}X^{2}+A_{3}^{t}X^{3}+A_{4}^{t}X^{4}\right)\kappa_{t}$$

where

$$A_{0}^{t}=A_{00}^{t}+A_{01}^{t}Y+A_{02}^{t}Y^{2}+A_{03}^{t}Y^{3}+A_{04}^{t}Y^{4}$$

$$A_{00}^{t}=2.031-0.635\theta -0.098\theta^{4}$$

$$A_{01}^{t}=2.525-3.464\theta +0.818\theta^{4}$$

$$A_{02}^{t}=-23.741+37.377\theta -10.314\theta^{4}$$

$$A_{03}^{t}=44.889-87.433\theta +28.653\theta^{4}$$

$$A_{04}^{t}=-26.097+60.519\theta -22.554\theta^{4}$$

$$A_{1}^{t}=A_{10}^{t}+A_{11}^{t}Y+A_{12}^{t}Y^{2}+A_{13}^{t}Y^{3}+A_{14}^{t}Y^{4}$$

$$A_{10}^{t}=-0.137-0.766\theta +0.877\theta^{4}$$

$$A_{11}^{t}=-0.159+0.794\theta +0.231\theta^{4}$$

$$A_{12}^{t}=7.915-17.261\theta +2.065\theta^{4}$$

$$A_{13}^{t}=-22.898+53.617\theta -17.877\theta^{4}$$

$$A_{14}^{t}=5.164-41.774\theta^{2}+30.281\theta^{4}$$

$$A_{2}^{t}=A_{20}^{t}+A_{21}^{t}Y+A_{22}^{t}Y^{2}+A_{23}^{t}Y^{3}+A_{24}^{t}Y^{4}$$

$$A_{20}^{t}=-0.545+3.275\theta^{2}-2.551\theta^{4}$$

$$A_{21}^{t}=-3.743+7.793\theta^{2}-5.112\theta^{4}$$

$$A_{22}^{t}=17.111-52.62\theta^{2}+39.271\theta^{4}$$

$$A_{23}^{t}=-19.451+73.353\theta^{2}-58.512\theta^{4}$$

$$A_{24}^{t}=6.94-32.1\theta^{2}+26.732\theta^{4}$$

$$A_{3}^{t}=A_{30}^{t}+A_{31}^{t}Y+A_{32}^{t}Y^{2}+A_{33}^{t}Y^{3}+A_{34}^{t}Y^{4}$$

$$A_{30}^{t}=0.198-3.351\theta^{2}+2.74\theta^{4}$$

$$A_{31}^{t}=7.209-13.931\theta^{2}+7.985\theta^{4}$$

$$A_{32}^{t}=-38.74+114.409\theta^{2}-69.799\theta^{4}$$

$$A_{33}^{t}=70.504-266.94\theta^{2}+155.111\theta^{4}$$

$$A_{34}^{t}=-53.268+231.865\theta^{2}-126.124\theta^{4}$$

$$A_{4}^{t}=A_{40}^{t}+A_{41}^{t}Y+A_{42}^{t}Y^{2}+A_{43}^{t}Y^{3}+A_{44}^{t}Y^{4}$$

$$A_{40}^{t}=4.321-15.621\theta +16.874\theta^{2}-5.485\theta^{4}$$

$$A_{41}^{t}=-5.321+12.0\theta^{2}-5.31\theta^{4}$$

$$A_{42}^{t}=28.713-110.239\theta^{2}+48.946\theta^{4}$$

$$A_{43}^{t}=-58.904+306.19\theta^{2}-131.962\theta^{4}$$

$$A_{44}^{t}=53.279-285.273\theta^{2}+122.094\theta^{4}$$

$$\begin{array}{l} \kappa_{t}=1+\left(\sqrt{Z}-1\right)\left\{1-\left[\left(-0.866+2.143\theta -0.452\theta^{2}\right)+\left(2.49+15.12\theta -9.25\theta^{2}\right)Y^{2}\right]X\right\}\cdot \\ \;\;\cdot Exp\left\{-{\left[\left(9.675-11.567\theta +4.966\theta^{2}\right)Y\right]}^{2.7}-\left(3.61-4.05\theta +1.66\theta^{2}\right)\right\} \end{array}$$

Bending load

(A2) $$K_{tb}=X^{n}\left(A_{0}^{b}+A_{1}^{b}X+A_{2}^{b}X^{2}+A_{3}^{b}X^{3}+A_{4}^{b}X^{4}\right)\kappa_{b}$$

where

$$A_{0}^{b}=A_{00}^{b}+A_{01}^{b}Y+A_{02}^{b}Y^{2}+A_{03}^{b}Y^{3}+A_{04}^{b}Y^{4}$$

$$A_{00}^{b}=1.904-0.751\theta^{2}+0.132\theta^{4}$$

$$A_{01}^{b}=-0.682+4.496\theta^{2}-2.507\theta^{4}$$

$$A_{02}^{b}=-13.6-7.777\theta^{2}+9.199\theta^{4}$$

$$A_{03}^{b}=41.72-12.838\theta^{2}-7.467\theta^{4}$$

$$A_{04}^{b}=-35.686+24.313\theta^{2}-1.331\theta^{4}$$

$$A_{1}^{b}=A_{10}^{b}+A_{11}^{b}Y+A_{12}^{b}Y^{2}+A_{13}^{b}Y^{3}+A_{14}^{b}Y^{4}$$

$$A_{10}^{b}=-0.098-0.801\theta +0.916\theta^{4}$$

$$A_{11}^{b}=0.767-1.604\theta -0.888\theta^{4}$$

$$A_{12}^{b}=-0.489+11.177\theta -8.132\theta^{4}$$

$$A_{13}^{b}=-8.508-10.312\theta +24.849\theta^{4}$$

$$A_{14}^{b}=10.8+0.608\theta^{2}-19.056\theta^{4}$$

$$A_{2}^{b}=A_{20}^{b}+A_{21}^{b}Y+A_{22}^{b}Y^{2}+A_{23}^{b}Y^{3}+A_{24}^{b}Y^{4}$$

$$A_{20}^{b}=-0.797+3.839\theta^{2}-2.973\theta^{4}$$

$$A_{21}^{b}=2.411-14.024\theta^{2}+12.923\theta^{4}$$

$$A_{22}^{b}=-0.091+17.933\theta^{2}-23.494\theta^{4}$$

$$A_{23}^{b}=-13.593+25.528\theta^{2}+5.148\theta^{4}$$

$$A_{24}^{b}=15.525-23.097\theta^{2}-6.363\theta^{4}$$

$$A_{3}^{b}=A_{30}^{b}+A_{31}^{b}Y+A_{32}^{b}Y^{2}+A_{33}^{b}Y^{3}+A_{34}^{b}Y^{4}$$

$$A_{30}^{b}=0.664-4.494\theta^{2}+3.458\theta^{4}$$

$$A_{31}^{b}=-4.748+24.121\theta^{2}-19.574\theta^{4}$$

$$A_{32}^{b}=7.857-67.243\theta^{2}+55.928\theta^{4}$$

$$A_{33}^{b}=2.829+135.344\theta^{2}-117.075\theta^{4}$$

$$A_{34}^{b}=5.16-195.991\theta^{2}+157.608\theta^{4}$$

$$A_{4}^{b}=A_{40}^{b}+A_{41}^{b}Y+A_{42}^{b}Y^{2}+A_{43}^{b}Y^{3}+A_{44}^{b}Y^{4}$$

$$A_{40}^{b}=-0.381+2.717\theta^{2}-2.003\theta^{4}$$

$$A_{41}^{b}=1.613-14.683\theta^{2}+9.871\theta^{4}$$

$$A_{42}^{b}=3.506+57.253\theta^{2}-35.678\theta^{4}$$

$$A_{43}^{b}=-3.197-194.43\theta^{2}+126.77\theta^{4}$$

$$A_{44}^{b}=-21.977+268.935\theta^{2}-178.897\theta^{4}$$

$$\begin{array}{l} \kappa_{b}=1+\left(\sqrt{Z}-1\right)\left\{1-\left[\left(-0.73+1.85\theta -0.2\theta^{2}\right)+\left(5.28+37.34\theta -23.44\theta^{2}\right)Y^{2}\right]X\right\}\cdot \\ \;\;\cdot Exp\left\{-{\left[\left(16.73-19.24\theta +8.56\theta^{2}\right)Y\right]}^{2.0}-\left(3.86-4.35\theta +1.7\theta^{2}\right)\right\} \end{array}$$

Shearing load

(A3) $$K_{ts}=X^{n_{s}}\left(A_{0}^{s}+A_{1}^{s}X+A_{2}^{s}X^{2}+A_{3}^{s}X^{3}+A_{4}^{s}X^{4}\right)\kappa_{s}$$

where

$$A_{0}^{s}=A_{00}^{s}+A_{01}^{s}Y^{2}+A_{02}^{s}Y^{3}+A_{03}^{s}Y^{4}$$

$$A_{00}^{s}=1.3265+0.3143\theta -0.3\theta^{2}$$

$$A_{01}^{s}=-7.2+5.91\theta -1.8355\theta^{2}$$

$$A_{02}^{s}=15.53-28.5882\theta^{2}+17.0\theta^{3}$$

$$A_{03}^{s}=-11.6+18.3\theta^{2}-8.1207\theta^{4}$$

$$A_{1}^{s}=-0.178-0.1095\theta^{2}+\left(0.1422+0.2533\theta^{2}\right)Y^{2}$$

$$A_{2}^{s}=A_{20}^{s}+A_{21}^{s}Y+A_{22}^{s}Y^{2}$$

$$A_{20}^{s}=2.9022-8.3702\theta +5.5285\theta^{2}$$

$$A_{21}^{s}=1.0-1.3728\theta^{2}$$

$$A_{22}^{s}=-2.8318+5.1835\theta^{2}$$

$$A_{3}^{s}=A_{30}^{s}+A_{31}^{s}Y+A_{32}^{s}Y^{3}$$

$$A_{30}^{s}=-7.88+21.2254\theta -13.2803\theta^{2}$$

$$A_{31}^{s}=-7.0+18.1182\theta -12.0\theta^{2}$$

$$A_{32}^{s}=11.7633-11.965\theta^{2}$$

$$A_{4}^{s}=A_{40}^{s}+A_{41}^{s}Y+A_{42}^{s}Y^{2}+A_{43}^{s}Y^{3}$$

$$A_{40}^{s}=8.1273-25.1758\theta +22.4116\theta^{2}-5.4681\theta^{3}$$

$$A_{41}^{s}=1.6654-17.8143\theta^{2}+17.908\theta^{3}$$

$$A_{42}^{s}=8.7364-11.4037\theta^{3}$$

$$A_{43}^{s}=-18.3578+19.0842\theta^{3}$$

$$\begin{array}{l} \kappa_{s}=1+\left(\sqrt{Z}-1\right)\left\{1-\left[\left(-0.40+0.67\theta +0.70\theta^{2}\right)+\left(-4.17+18.54\theta -6.94\theta^{2}\right){\left(\frac{2\cdot Y}{1+Y}\right)}^{2}\right]X^{2}\right\}\cdot \\ \;\;\cdot Exp\left\{-{\left[\left(6.26-5.74\theta +2.52\theta^{2}\right)\frac{2\cdot Y}{1+Y}\right]}^{2.0}-\left(3.84-3.31\theta +1.23\theta^{2}\right)\right\} \end{array}$$

## Appendix B

Numerical SCF results for various weld angles *θ* for a cruciform joint subjected to tensile, bending, and shearing loads, while *T*/*a* = 1.

**Table A1:** *K_tt_* values for a cruciform joint, while *θ* = 30° and *T*/*a* = 1. Tensile load.

*θ* = 30°	*X*															
*Y*	0.010	0.018	0.025	0.032	0.050	0.079	0.100	0.150	0.200	0.250	0.300	0.350	0.400	0.450	0.500	0.562
**0.010**	5.323	4.598	4.215	3.967	3.522	3.115	2.927	2.611	2.398	2.237	2.106	1.996	1.899	1.813	1.735	1.645
**0.018**	5.324	4.600	4.216	3.969	3.523	3.116	2.927	2.612	2.399	2.237	2.107	1.996	1.900	1.814	1.735	1.645
**0.025**	5.324	4.602	4.217	3.971	3.525	3.117	2.928	2.613	2.400	2.238	2.108	1.997	1.900	1.814	1.736	1.646
**0.032**	5.326	4.604	4.220	3.972	3.526	3.119	2.930	2.614	2.401	2.239	2.108	1.998	1.901	1.815	1.737	1.647
**0.050**	5.326	4.603	4.221	3.974	3.530	3.122	2.933	2.618	2.404	2.243	2.112	2.001	1.904	1.818	1.739	1.649
**0.079**	5.342	4.618	4.235	3.986	3.541	3.132	2.943	2.626	2.411	2.249	2.118	2.007	1.910	1.823	1.744	1.653
**0.100**	5.347	4.625	4.240	3.991	3.545	3.136	2.946	2.629	2.414	2.252	2.120	2.009	1.912	1.824	1.745	1.654
**0.150**	5.318	4.597	4.214	3.967	3.523	3.117	2.929	2.612	2.399	2.237	2.106	1.994	1.897	1.810	1.730	1.638
**0.200**	5.199	4.492	4.118	3.877	3.444	3.046	2.862	2.552	2.344	2.185	2.056	1.946	1.850	1.764	1.686	1.593
**0.250**	5.009	4.326	3.967	3.734	3.316	2.933	2.755	2.458	2.256	2.102	1.978	1.872	1.779	1.695	1.618	1.529
**0.300**	4.786	4.133	3.786	3.565	3.166	2.800	2.630	2.346	2.153	2.006	1.887	1.786	1.697	1.617	1.543	1.458
**0.350**	4.543	3.930	3.601	3.390	3.010	2.662	2.500	2.230	2.047	1.907	1.794	1.698	1.614	1.538	1.468	1.388
**0.400**	4.327	3.732	3.420	3.219	2.859	2.528	2.375	2.118	1.944	1.812	1.705	1.614	1.534	1.463	1.398	1.324
**0.450**	4.108	3.548	3.250	3.060	2.716	2.402	2.256	2.013	1.848	1.723	1.621	1.536	1.461	1.395	1.334	1.267
**0.500**	3.912	3.372	3.089	2.908	2.582	2.283	2.145	1.914	1.757	1.639	1.543	1.463	1.393	1.332	1.278	1.218
**0.562**	3.670	3.166	2.902	2.730	2.423	2.144	2.014	1.797	1.652	1.542	1.454	1.380	1.318	1.264	1.217	1.168

**Table A2:** *K_tt_* values for a cruciform joint, while *θ* = 40° and *T*/*a* = 1. Tensile load.

*θ* = 40°	*X*															
*Y*	0.010	0.018	0.025	0.032	0.050	0.079	0.100	0.150	0.200	0.250	0.300	0.350	0.400	0.450	0.500	0.562
**0.010**	6.365	5.322	4.791	4.452	3.853	3.322	3.081	2.690	2.434	2.246	2.098	1.976	1.873	1.783	1.702	1.614
**0.018**	6.366	5.324	4.792	4.453	3.854	3.323	3.082	2.691	2.435	2.247	2.098	1.977	1.873	1.783	1.703	1.614
**0.025**	6.368	5.326	4.793	4.454	3.855	3.324	3.083	2.691	2.436	2.247	2.099	1.977	1.874	1.783	1.703	1.615
**0.032**	6.369	5.325	4.795	4.455	3.856	3.324	3.084	2.692	2.436	2.248	2.100	1.978	1.874	1.784	1.704	1.615
**0.050**	6.363	5.327	4.795	4.456	3.858	3.327	3.087	2.695	2.439	2.251	2.102	1.980	1.877	1.786	1.706	1.617
**0.079**	6.384	5.346	4.809	4.470	3.870	3.338	3.096	2.704	2.447	2.258	2.109	1.986	1.882	1.791	1.711	1.621
**0.100**	6.398	5.359	4.821	4.480	3.879	3.345	3.103	2.709	2.452	2.262	2.113	1.990	1.885	1.794	1.713	1.623
**0.150**	6.415	5.369	4.830	4.489	3.887	3.351	3.109	2.713	2.454	2.263	2.113	1.989	1.883	1.791	1.708	1.616
**0.200**	6.353	5.318	4.784	4.446	3.849	3.319	3.078	2.685	2.427	2.237	2.086	1.962	1.856	1.762	1.679	1.584
**0.250**	6.203	5.193	4.672	4.342	3.759	3.239	3.004	2.619	2.366	2.179	2.031	1.908	1.803	1.710	1.626	1.532
**0.300**	5.983	5.009	4.506	4.188	3.624	3.123	2.895	2.524	2.279	2.097	1.953	1.833	1.731	1.640	1.559	1.466
**0.350**	5.718	4.787	4.305	4.001	3.463	2.984	2.766	2.410	2.175	2.001	1.863	1.748	1.649	1.563	1.485	1.397
**0.400**	5.433	4.546	4.090	3.801	3.289	2.834	2.627	2.289	2.065	1.900	1.769	1.660	1.566	1.484	1.411	1.330
**0.450**	5.140	4.302	3.870	3.596	3.112	2.681	2.485	2.166	1.955	1.798	1.675	1.572	1.485	1.409	1.342	1.270
**0.500**	4.853	4.060	3.653	3.394	2.938	2.531	2.346	2.045	1.846	1.700	1.584	1.489	1.409	1.340	1.280	1.217
**0.562**	4.506	3.769	3.390	3.150	2.726	2.350	2.178	1.900	1.717	1.583	1.478	1.393	1.323	1.264	1.215	1.166

**Table A3:** *K_tt_* values for a cruciform joint, while *θ* = 50° and *T*/*a* = 1. Tensile load.

*θ* = 50°	*X*															
*Y*	0.010	0.018	0.025	0.032	0.050	0.079	0.100	0.150	0.200	0.250	0.300	0.350	0.400	0.450	0.500	0.562
**0.010**	7.054	5.749	5.097	4.688	3.982	3.373	3.103	2.676	2.405	2.211	2.061	1.941	1.840	1.754	1.678	1.596
**0.018**	7.055	5.750	5.097	4.689	3.983	3.373	3.104	2.676	2.405	2.211	2.061	1.941	1.840	1.754	1.679	1.596
**0.025**	7.056	5.751	5.099	4.690	3.984	3.374	3.104	2.677	2.406	2.211	2.062	1.941	1.841	1.754	1.679	1.597
**0.032**	7.059	5.753	5.100	4.691	3.985	3.375	3.105	2.678	2.406	2.212	2.062	1.942	1.841	1.755	1.680	1.597
**0.050**	7.042	5.749	5.098	4.691	3.986	3.377	3.108	2.680	2.409	2.214	2.065	1.944	1.843	1.757	1.681	1.599
**0.079**	7.065	5.766	5.113	4.704	3.997	3.387	3.116	2.687	2.415	2.220	2.070	1.949	1.848	1.761	1.686	1.603
**0.100**	7.084	5.780	5.126	4.716	4.007	3.395	3.124	2.693	2.421	2.225	2.074	1.953	1.851	1.764	1.688	1.605
**0.150**	7.121	5.811	5.151	4.740	4.027	3.411	3.138	2.705	2.429	2.232	2.079	1.956	1.853	1.764	1.686	1.600
**0.200**	7.105	5.800	5.142	4.730	4.018	3.402	3.128	2.694	2.417	2.218	2.064	1.939	1.834	1.743	1.663	1.574
**0.250**	7.012	5.724	5.074	4.668	3.963	3.353	3.082	2.651	2.376	2.177	2.023	1.897	1.792	1.700	1.618	1.526
**0.300**	6.837	5.579	4.943	4.548	3.860	3.264	2.999	2.577	2.306	2.110	1.958	1.834	1.729	1.638	1.556	1.465
**0.350**	6.588	5.376	4.763	4.381	3.718	3.143	2.887	2.478	2.215	2.025	1.877	1.756	1.654	1.565	1.486	1.398
**0.400**	6.296	5.136	4.551	4.186	3.551	3.000	2.755	2.363	2.111	1.929	1.787	1.671	1.573	1.488	1.413	1.331
**0.450**	5.975	4.873	4.318	3.971	3.368	2.845	2.612	2.240	2.001	1.827	1.693	1.583	1.491	1.412	1.344	1.270
**0.500**	5.640	4.599	4.075	3.747	3.178	2.685	2.465	2.114	1.888	1.725	1.599	1.497	1.413	1.342	1.281	1.217
**0.562**	5.214	4.251	3.766	3.464	2.938	2.483	2.280	1.956	1.749	1.601	1.487	1.397	1.323	1.263	1.214	1.165

**Table A4:** *K_tt_* values for a cruciform joint, while *θ* = 60° and *T*/*a* = 1. Tensile load.

*θ* = 60°	*X*															
*Y*	0.010	0.018	0.025	0.032	0.050	0.079	0.100	0.150	0.200	0.250	0.300	0.350	0.400	0.450	0.500	0.562
**0.010**	7.381	5.922	5.194	4.746	3.984	3.343	3.065	2.633	2.364	2.175	2.031	1.916	1.821	1.741	1.671	1.595
**0.018**	7.403	5.923	5.196	4.747	3.984	3.343	3.066	2.633	2.365	2.175	2.032	1.917	1.822	1.741	1.671	1.595
**0.025**	7.405	5.925	5.196	4.748	3.985	3.344	3.066	2.634	2.365	2.176	2.032	1.917	1.822	1.741	1.671	1.596
**0.032**	7.407	5.924	5.198	4.749	3.986	3.344	3.067	2.634	2.366	2.176	2.033	1.918	1.822	1.742	1.672	1.596
**0.050**	7.392	5.920	5.195	4.748	3.987	3.347	3.068	2.636	2.368	2.179	2.034	1.920	1.824	1.744	1.674	1.598
**0.079**	7.409	5.936	5.208	4.761	3.998	3.355	3.077	2.643	2.374	2.184	2.039	1.924	1.829	1.748	1.678	1.602
**0.100**	7.427	5.950	5.220	4.772	4.007	3.363	3.083	2.648	2.378	2.188	2.043	1.928	1.832	1.751	1.680	1.604
**0.150**	7.479	5.988	5.254	4.801	4.031	3.382	3.100	2.661	2.389	2.196	2.050	1.932	1.835	1.752	1.679	1.599
**0.200**	7.496	6.001	5.265	4.810	4.037	3.384	3.101	2.658	2.383	2.188	2.039	1.919	1.819	1.733	1.657	1.573
**0.250**	7.449	5.964	5.229	4.777	4.006	3.355	3.071	2.629	2.352	2.155	2.005	1.883	1.781	1.693	1.614	1.526
**0.300**	7.319	5.857	5.135	4.690	3.930	3.287	3.007	2.569	2.294	2.098	1.948	1.826	1.723	1.634	1.554	1.465
**0.350**	7.107	5.688	4.986	4.552	3.812	3.185	2.912	2.483	2.213	2.021	1.873	1.752	1.651	1.563	1.485	1.398
**0.400**	6.841	5.469	4.793	4.375	3.662	3.057	2.793	2.378	2.117	1.931	1.788	1.670	1.572	1.488	1.414	1.331
**0.450**	6.524	5.215	4.570	4.171	3.489	2.911	2.658	2.261	2.011	1.833	1.695	1.585	1.493	1.413	1.344	1.270
**0.500**	6.178	4.937	4.326	3.948	3.302	2.753	2.514	2.137	1.900	1.732	1.602	1.499	1.414	1.342	1.281	1.217
**0.562**	5.719	4.571	4.004	3.655	3.056	2.548	2.326	1.978	1.760	1.606	1.489	1.397	1.324	1.263	1.214	1.165

**Table A5:** *K_tb_* values for a cruciform joint, while *θ* = 30° and *T*/*a* = 1. Bending load.

*θ* = 30°	*X*															
*Y*	0.010	0.018	0.025	0.032	0.050	0.079	0.100	0.150	0.200	0.250	0.300	0.350	0.400	0.450	0.500	0.562
**0.010**	5.322	4.600	4.218	3.971	3.527	3.121	2.931	2.616	2.402	2.241	2.110	1.999	1.903	1.816	1.737	1.648
**0.018**	5.326	4.603	4.221	3.974	3.530	3.123	2.933	2.617	2.405	2.242	2.111	2.000	1.903	1.817	1.738	1.647
**0.025**	5.324	4.603	4.221	3.973	3.529	3.122	2.933	2.617	2.404	2.242	2.110	2.000	1.903	1.816	1.737	1.646
**0.032**	5.320	4.600	4.217	3.970	3.526	3.120	2.931	2.615	2.401	2.240	2.109	1.998	1.901	1.814	1.735	1.644
**0.050**	5.295	4.576	4.196	3.951	3.509	3.105	2.916	2.602	2.389	2.228	2.098	1.987	1.890	1.804	1.725	1.634
**0.079**	5.193	4.488	4.116	3.875	3.441	3.044	2.860	2.551	2.343	2.185	2.056	1.948	1.853	1.767	1.689	1.600
**0.100**	5.087	4.399	4.033	3.797	3.372	2.983	2.802	2.500	2.296	2.140	2.015	1.908	1.815	1.731	1.654	1.566
**0.150**	4.772	4.125	3.781	3.560	3.162	2.797	2.628	2.345	2.153	2.008	1.890	1.790	1.703	1.625	1.553	1.471
**0.200**	4.448	3.842	3.523	3.317	2.946	2.606	2.449	2.185	2.008	1.873	1.763	1.672	1.591	1.519	1.454	1.380
**0.250**	4.159	3.592	3.294	3.101	2.754	2.437	2.290	2.045	1.879	1.754	1.653	1.569	1.495	1.430	1.372	1.306
**0.300**	3.908	3.375	3.093	2.912	2.590	2.292	2.151	1.925	1.770	1.654	1.558	1.481	1.414	1.356	1.304	1.247
**0.350**	3.685	3.189	2.922	2.751	2.444	2.164	2.034	1.818	1.674	1.566	1.480	1.409	1.348	1.296	1.250	1.201
**0.400**	3.504	3.023	2.771	2.609	2.318	2.053	1.930	1.727	1.592	1.492	1.413	1.348	1.294	1.247	1.208	1.165
**0.450**	3.333	2.880	2.638	2.485	2.208	1.956	1.840	1.649	1.522	1.429	1.357	1.298	1.250	1.209	1.174	1.138
**0.500**	3.170	2.733	2.505	2.359	2.097	1.859	1.749	1.570	1.452	1.367	1.301	1.249	1.206	1.171	1.141	1.111
**0.562**	2.974	2.567	2.354	2.216	1.970	1.749	1.647	1.482	1.376	1.300	1.243	1.198	1.162	1.133	1.110	1.085

**Table A6:** *K_tb_* values for a cruciform joint, while *θ* = 40° and *T*/*a* = 1. Bending load.

*θ* = 40°	*X*															
*Y*	0.010	0.018	0.025	0.032	0.050	0.079	0.100	0.150	0.200	0.250	0.300	0.350	0.400	0.450	0.500	0.562
**0.010**	6.370	5.331	4.799	4.460	3.862	3.330	3.089	2.697	2.440	2.252	2.103	1.981	1.876	1.786	1.706	1.616
**0.018**	6.380	5.341	4.807	4.468	3.868	3.336	3.094	2.701	2.444	2.255	2.106	1.983	1.879	1.787	1.706	1.617
**0.025**	6.387	5.347	4.812	4.472	3.872	3.339	3.097	2.704	2.446	2.256	2.107	1.984	1.879	1.788	1.707	1.617
**0.032**	6.391	5.350	4.815	4.475	3.874	3.341	3.099	2.705	2.447	2.257	2.107	1.983	1.879	1.787	1.706	1.616
**0.050**	6.388	5.347	4.812	4.472	3.872	3.339	3.096	2.702	2.444	2.253	2.103	1.979	1.874	1.782	1.700	1.609
**0.079**	6.323	5.294	4.763	4.427	3.832	3.304	3.063	2.673	2.416	2.227	2.078	1.954	1.849	1.757	1.675	1.583
**0.100**	6.239	5.224	4.700	4.367	3.781	3.259	3.022	2.636	2.382	2.195	2.047	1.925	1.821	1.729	1.648	1.557
**0.150**	5.930	4.963	4.465	4.149	3.592	3.095	2.870	2.503	2.262	2.083	1.942	1.825	1.726	1.638	1.560	1.473
**0.200**	5.543	4.640	4.174	3.879	3.358	2.894	2.684	2.341	2.115	1.949	1.818	1.709	1.617	1.536	1.465	1.385
**0.250**	5.156	4.317	3.884	3.609	3.125	2.694	2.499	2.181	1.972	1.819	1.698	1.599	1.516	1.443	1.380	1.310
**0.300**	4.799	4.016	3.614	3.364	2.913	2.512	2.331	2.033	1.844	1.701	1.591	1.504	1.429	1.365	1.310	1.248
**0.350**	4.487	3.754	3.378	3.139	2.719	2.347	2.179	1.906	1.729	1.601	1.502	1.422	1.356	1.300	1.252	1.201
**0.400**	4.211	3.522	3.169	2.946	2.552	2.204	2.048	1.795	1.633	1.516	1.426	1.355	1.297	1.248	1.208	1.165
**0.450**	3.966	3.317	2.984	2.774	2.405	2.080	1.934	1.700	1.550	1.444	1.364	1.301	1.251	1.209	1.174	1.138
**0.500**	3.724	3.115	2.803	2.606	2.261	1.958	1.822	1.606	1.471	1.376	1.305	1.250	1.206	1.170	1.141	1.111
**0.562**	3.451	2.886	2.598	2.416	2.099	1.821	1.698	1.505	1.385	1.303	1.243	1.198	1.162	1.133	1.109	1.085

**Table A7:** *K_tb_* values for a cruciform joint, while *θ* = 50° and *T*/*a* = 1. Bending load.

*θ* = 50°	*X*															
*Y*	0.010	0.018	0.025	0.032	0.050	0.079	0.100	0.150	0.200	0.250	0.300	0.350	0.400	0.450	0.500	0.562
**0.010**	7.062	5.762	5.109	4.701	3.994	3.383	3.113	2.684	2.412	2.217	2.066	1.945	1.844	1.757	1.681	1.598
**0.018**	7.080	5.777	5.122	4.713	4.004	3.391	3.120	2.690	2.416	2.220	2.069	1.948	1.846	1.758	1.682	1.599
**0.025**	7.094	5.789	5.132	4.722	4.012	3.397	3.126	2.694	2.420	2.223	2.071	1.949	1.847	1.759	1.683	1.599
**0.032**	7.105	5.798	5.141	4.729	4.017	3.402	3.129	2.697	2.422	2.224	2.072	1.949	1.847	1.759	1.682	1.598
**0.050**	7.121	5.813	5.154	4.742	4.027	3.409	3.135	2.700	2.424	2.225	2.072	1.948	1.845	1.756	1.678	1.593
**0.079**	7.099	5.792	5.134	4.723	4.011	3.394	3.120	2.685	2.408	2.208	2.055	1.930	1.826	1.736	1.657	1.571
**0.100**	7.044	5.746	5.094	4.686	3.978	3.365	3.093	2.660	2.384	2.186	2.032	1.907	1.803	1.713	1.634	1.547
**0.150**	6.780	5.531	4.902	4.509	3.826	3.235	2.972	2.553	2.286	2.093	1.944	1.823	1.720	1.632	1.555	1.469
**0.200**	6.390	5.215	4.621	4.250	3.607	3.049	2.801	2.405	2.153	1.970	1.829	1.716	1.619	1.537	1.464	1.385
**0.250**	5.958	4.862	4.309	3.963	3.363	2.844	2.613	2.245	2.010	1.842	1.712	1.607	1.520	1.445	1.380	1.310
**0.300**	5.528	4.517	4.002	3.682	3.127	2.644	2.427	2.092	1.876	1.719	1.604	1.510	1.430	1.364	1.308	1.250
**0.350**	5.141	4.190	3.714	3.417	2.902	2.458	2.261	1.950	1.753	1.614	1.509	1.425	1.357	1.300	1.252	1.201
**0.400**	4.782	3.901	3.458	3.183	2.705	2.294	2.113	1.827	1.649	1.524	1.430	1.356	1.297	1.248	1.207	1.164
**0.450**	4.473	3.645	3.232	2.975	2.532	2.150	1.984	1.723	1.561	1.449	1.366	1.301	1.250	1.208	1.174	1.138
**0.500**	4.160	3.394	3.011	2.773	2.362	2.011	1.858	1.621	1.476	1.377	1.305	1.249	1.206	1.170	1.141	1.111
**0.562**	3.814	3.112	2.762	2.546	2.174	1.858	1.721	1.512	1.387	1.303	1.243	1.197	1.161	1.133	1.109	1.085

**Table A8:** *K_tb_* values for a cruciform joint, while *θ* = 60° and *T*/*a* = 1. Bending load.

*θ* = 60°	*X*															
*Y*	0.010	0.018	0.025	0.032	0.050	0.079	0.100	0.150	0.200	0.250	0.300	0.350	0.400	0.450	0.500	0.562
**0.010**	7.417	5.938	5.211	4.762	3.999	3.355	3.076	2.642	2.372	2.181	2.036	1.921	1.825	1.744	1.673	1.597
**0.018**	7.442	5.959	5.229	4.778	4.011	3.365	3.084	2.648	2.376	2.185	2.040	1.923	1.827	1.745	1.675	1.598
**0.025**	7.464	5.976	5.242	4.791	4.021	3.372	3.091	2.652	2.380	2.188	2.042	1.925	1.828	1.746	1.675	1.598
**0.032**	7.481	5.989	5.254	4.801	4.029	3.378	3.096	2.656	2.382	2.190	2.043	1.926	1.829	1.746	1.675	1.597
**0.050**	7.521	6.021	5.281	4.825	4.047	3.392	3.107	2.663	2.387	2.192	2.044	1.925	1.827	1.744	1.671	1.592
**0.079**	7.532	6.029	5.287	4.829	4.048	3.389	3.103	2.656	2.377	2.180	2.030	1.910	1.810	1.725	1.651	1.570
**0.100**	7.504	6.006	5.265	4.809	4.030	3.372	3.085	2.638	2.360	2.162	2.011	1.891	1.790	1.704	1.629	1.546
**0.150**	7.307	5.844	5.122	4.675	3.915	3.271	2.990	2.551	2.277	2.082	1.933	1.814	1.714	1.628	1.552	1.469
**0.200**	6.953	5.560	4.872	4.447	3.721	3.106	2.838	2.418	2.156	1.970	1.827	1.712	1.617	1.535	1.464	1.385
**0.250**	6.517	5.212	4.566	4.168	3.487	2.910	2.659	2.265	2.019	1.846	1.713	1.607	1.519	1.445	1.380	1.310
**0.300**	6.058	4.847	4.247	3.877	3.244	2.705	2.472	2.112	1.886	1.727	1.606	1.508	1.432	1.367	1.308	1.250
**0.350**	5.611	4.487	3.932	3.591	3.006	2.514	2.300	1.967	1.761	1.618	1.510	1.425	1.357	1.300	1.251	1.201
**0.400**	5.201	4.160	3.647	3.331	2.793	2.339	2.143	1.840	1.654	1.526	1.429	1.356	1.297	1.248	1.207	1.164
**0.450**	4.838	3.867	3.392	3.099	2.602	2.185	2.006	1.730	1.563	1.449	1.365	1.301	1.250	1.208	1.174	1.138
**0.500**	4.475	3.581	3.143	2.874	2.418	2.037	1.874	1.626	1.477	1.377	1.304	1.249	1.205	1.170	1.141	1.111
**0.562**	4.067	3.259	2.864	2.621	2.212	1.874	1.730	1.514	1.387	1.303	1.242	1.197	1.161	1.133	1.109	1.085

**Table A9:** *K_ts_* values for a cruciform joint, while *θ* = 30° and *T*/*a* = 1. Shearing load.

*θ* = 30°	*X*															
*Y*	0.010	0.018	0.025	0.032	0.050	0.079	0.100	0.150	0.200	0.250	0.300	0.350	0.400	0.450	0.500	0.562
**0.010**	2.705	2.493	2.372	2.292	2.142	1.996	1.925	1.802	1.715	1.647	1.589	1.539	1.494	1.453	1.414	1.367
**0.018**	2.704	2.491	2.371	2.291	2.140	1.995	1.924	1.801	1.714	1.646	1.588	1.538	1.493	1.452	1.413	1.367
**0.025**	2.702	2.489	2.369	2.290	2.139	1.993	1.923	1.800	1.713	1.645	1.587	1.537	1.492	1.451	1.412	1.366
**0.032**	2.698	2.486	2.367	2.287	2.137	1.991	1.921	1.798	1.711	1.643	1.586	1.536	1.491	1.449	1.410	1.364
**0.050**	2.683	2.514	2.360	2.277	2.129	1.984	1.914	1.791	1.705	1.637	1.580	1.530	1.485	1.444	1.405	1.359
**0.079**	2.657	2.451	2.334	2.257	2.108	1.964	1.895	1.774	1.689	1.621	1.565	1.515	1.471	1.430	1.391	1.346
**0.100**	2.640	2.432	2.315	2.236	2.089	1.947	1.878	1.758	1.673	1.607	1.551	1.502	1.458	1.417	1.379	1.334
**0.150**	2.568	2.365	2.251	2.175	2.032	1.894	1.827	1.710	1.628	1.563	1.508	1.461	1.418	1.379	1.342	1.298
**0.200**	2.487	2.291	2.180	2.106	1.968	1.834	1.769	1.656	1.576	1.513	1.461	1.415	1.374	1.336	1.301	1.259
**0.250**	2.407	2.216	2.110	2.038	1.904	1.775	1.712	1.603	1.525	1.465	1.414	1.370	1.331	1.295	1.261	1.222
**0.300**	2.331	2.146	2.043	1.973	1.844	1.718	1.658	1.552	1.478	1.419	1.370	1.328	1.291	1.257	1.225	1.188
**0.350**	2.259	2.081	1.981	1.913	1.788	1.666	1.607	1.505	1.433	1.377	1.330	1.290	1.254	1.222	1.193	1.159
**0.400**	2.193	2.020	1.922	1.857	1.735	1.617	1.560	1.462	1.392	1.338	1.293	1.255	1.221	1.191	1.164	1.134
**0.450**	2.130	1.962	1.867	1.804	1.686	1.572	1.516	1.421	1.354	1.302	1.259	1.223	1.192	1.164	1.139	1.112
**0.500**	2.071	1.907	1.815	1.754	1.639	1.528	1.474	1.382	1.317	1.268	1.227	1.194	1.165	1.139	1.117	1.094
**0.562**	1.998	1.840	1.751	1.692	1.581	1.475	1.423	1.335	1.274	1.228	1.190	1.160	1.134	1.112	1.094	1.074

**Table A10:** *K_ts_* values for a cruciform joint, while *θ* = 40° and *T*/*a* = 1. Shearing load.

*θ* = 40°	*X*															
*Y*	0.010	0.018	0.025	0.032	0.050	0.079	0.100	0.150	0.200	0.250	0.300	0.350	0.400	0.450	0.500	0.562
**0.010**	3.221	2.908	2.723	2.619	2.391	2.186	2.088	1.921	1.805	1.715	1.641	1.577	1.521	1.471	1.425	1.372
**0.018**	3.219	2.899	2.722	2.606	2.390	2.185	2.087	1.920	1.804	1.714	1.640	1.577	1.521	1.470	1.424	1.371
**0.025**	3.217	2.897	2.720	2.604	2.388	2.184	2.086	1.919	1.803	1.713	1.639	1.576	1.520	1.469	1.423	1.370
**0.032**	3.210	2.895	2.718	2.601	2.386	2.182	2.084	1.917	1.801	1.712	1.638	1.574	1.519	1.468	1.422	1.369
**0.050**	3.212	2.886	2.711	2.594	2.379	2.175	2.078	1.911	1.796	1.706	1.633	1.570	1.514	1.464	1.417	1.364
**0.079**	3.162	2.859	2.688	2.573	2.361	2.157	2.063	1.896	1.781	1.692	1.619	1.557	1.501	1.451	1.406	1.353
**0.100**	3.154	2.841	2.667	2.553	2.341	2.141	2.045	1.881	1.767	1.679	1.607	1.544	1.490	1.440	1.395	1.342
**0.150**	3.075	2.769	2.600	2.489	2.282	2.086	1.993	1.833	1.722	1.637	1.566	1.506	1.452	1.404	1.360	1.309
**0.200**	2.977	2.681	2.518	2.410	2.210	2.020	1.930	1.776	1.668	1.585	1.517	1.459	1.407	1.361	1.319	1.270
**0.250**	2.874	2.588	2.430	2.325	2.133	1.950	1.863	1.714	1.611	1.531	1.466	1.410	1.361	1.317	1.277	1.231
**0.300**	2.771	2.495	2.342	2.242	2.056	1.880	1.796	1.653	1.554	1.477	1.415	1.362	1.316	1.275	1.237	1.196
**0.350**	2.671	2.405	2.258	2.161	1.982	1.812	1.732	1.594	1.499	1.426	1.367	1.317	1.274	1.236	1.202	1.164
**0.400**	2.576	2.319	2.177	2.084	1.912	1.748	1.671	1.539	1.448	1.379	1.323	1.276	1.236	1.201	1.170	1.137
**0.450**	2.485	2.237	2.101	2.011	1.845	1.687	1.613	1.486	1.400	1.334	1.282	1.239	1.202	1.170	1.143	1.114
**0.500**	2.397	2.159	2.027	1.940	1.780	1.629	1.557	1.436	1.354	1.293	1.244	1.204	1.171	1.143	1.120	1.094
**0.562**	2.292	2.064	1.938	1.855	1.702	1.558	1.491	1.377	1.301	1.244	1.201	1.166	1.138	1.114	1.095	1.074

**Table A11:** *K_ts_* values for a cruciform joint, while *θ* = 50° and *T*/*a* = 1. Shearing load.

*θ* = 50°	*X*															
*Y*	0.010	0.018	0.025	0.032	0.050	0.079	0.100	0.150	0.200	0.250	0.300	0.350	0.400	0.450	0.500	0.562
**0.010**	3.716	3.275	3.038	2.882	2.600	2.337	2.213	2.005	1.863	1.755	1.669	1.596	1.533	1.478	1.428	1.373
**0.018**	3.713	3.273	3.036	2.881	2.598	2.335	2.212	2.004	1.862	1.755	1.668	1.595	1.532	1.477	1.427	1.372
**0.025**	3.709	3.271	3.035	2.880	2.597	2.334	2.211	2.003	1.861	1.754	1.667	1.594	1.532	1.476	1.427	1.371
**0.032**	3.707	3.268	3.032	2.878	2.595	2.333	2.209	2.001	1.860	1.753	1.666	1.593	1.531	1.475	1.426	1.370
**0.050**	3.701	3.262	3.023	2.870	2.587	2.326	2.203	1.996	1.854	1.748	1.661	1.589	1.526	1.471	1.421	1.366
**0.079**	3.667	3.265	3.001	2.849	2.569	2.309	2.186	1.981	1.841	1.734	1.649	1.577	1.515	1.460	1.410	1.355
**0.100**	3.647	3.215	2.981	2.829	2.552	2.293	2.172	1.967	1.828	1.723	1.637	1.566	1.504	1.449	1.400	1.345
**0.150**	3.561	3.139	2.911	2.762	2.491	2.239	2.120	1.920	1.785	1.682	1.598	1.528	1.468	1.414	1.366	1.312
**0.200**	3.450	3.041	2.820	2.676	2.413	2.169	2.054	1.861	1.729	1.630	1.549	1.482	1.423	1.372	1.326	1.274
**0.250**	3.325	2.931	2.719	2.580	2.326	2.091	1.980	1.794	1.668	1.572	1.496	1.431	1.376	1.327	1.283	1.235
**0.300**	3.197	2.818	2.613	2.480	2.237	2.010	1.904	1.726	1.605	1.514	1.441	1.380	1.328	1.283	1.242	1.198
**0.350**	3.070	2.706	2.509	2.381	2.148	1.931	1.829	1.659	1.544	1.458	1.389	1.332	1.284	1.242	1.205	1.166
**0.400**	2.946	2.597	2.408	2.286	2.062	1.854	1.757	1.594	1.485	1.404	1.340	1.287	1.243	1.205	1.173	1.138
**0.450**	2.827	2.492	2.311	2.193	1.979	1.780	1.687	1.533	1.430	1.354	1.295	1.246	1.207	1.173	1.145	1.115
**0.500**	2.711	2.390	2.216	2.104	1.898	1.709	1.621	1.474	1.378	1.307	1.253	1.210	1.174	1.145	1.120	1.095
**0.562**	2.570	2.266	2.102	1.995	1.802	1.623	1.541	1.405	1.317	1.254	1.206	1.169	1.139	1.115	1.095	1.074

**Table A12:** *K_ts_* values for a cruciform joint, while *θ* = 60° and *T*/*a* = 1. Shearing load.

*θ* = 60°	*X*															
*Y*	0.010	0.018	0.025	0.032	0.050	0.079	0.100	0.150	0.200	0.250	0.300	0.350	0.400	0.450	0.500	0.562
**0.010**	4.186	3.621	3.320	3.126	2.777	2.458	2.310	2.066	1.903	1.782	1.687	1.608	1.541	1.484	1.433	1.378
**0.018**	4.184	3.618	3.319	3.125	2.776	2.457	2.310	2.065	1.903	1.782	1.686	1.607	1.541	1.483	1.432	1.377
**0.025**	4.183	3.617	3.318	3.124	2.775	2.456	2.308	2.064	1.902	1.781	1.685	1.607	1.540	1.482	1.432	1.376
**0.032**	4.176	3.614	3.314	3.122	2.773	2.454	2.307	2.063	1.900	1.780	1.684	1.605	1.539	1.481	1.430	1.375
**0.050**	4.160	3.606	3.306	3.113	2.767	2.447	2.305	2.057	1.895	1.775	1.680	1.601	1.535	1.477	1.426	1.371
**0.079**	4.139	3.576	3.281	3.092	2.756	2.430	2.285	2.043	1.882	1.762	1.668	1.589	1.523	1.466	1.415	1.360
**0.100**	4.114	3.558	3.263	3.073	2.729	2.415	2.270	2.030	1.870	1.751	1.656	1.579	1.513	1.455	1.405	1.349
**0.150**	4.020	3.478	3.189	3.003	2.667	2.360	2.218	1.983	1.827	1.710	1.618	1.542	1.477	1.421	1.371	1.316
**0.200**	3.896	3.370	3.091	2.910	2.585	2.287	2.150	1.922	1.771	1.658	1.569	1.495	1.433	1.378	1.330	1.277
**0.250**	3.754	3.247	2.977	2.803	2.490	2.204	2.071	1.853	1.707	1.599	1.514	1.443	1.384	1.332	1.287	1.237
**0.300**	3.601	3.115	2.856	2.690	2.389	2.115	1.988	1.779	1.640	1.538	1.457	1.391	1.335	1.287	1.245	1.200
**0.350**	3.447	2.982	2.735	2.575	2.288	2.026	1.905	1.706	1.574	1.478	1.402	1.340	1.289	1.245	1.207	1.167
**0.400**	3.295	2.851	2.615	2.462	2.188	1.938	1.823	1.634	1.510	1.420	1.350	1.293	1.247	1.207	1.174	1.138
**0.450**	3.147	2.723	2.498	2.352	2.091	1.853	1.744	1.566	1.450	1.366	1.302	1.251	1.209	1.174	1.145	1.115
**0.500**	3.003	2.599	2.384	2.245	1.996	1.771	1.668	1.501	1.393	1.316	1.258	1.212	1.176	1.146	1.121	1.095
**0.562**	2.828	2.447	2.245	2.115	1.882	1.672	1.577	1.424	1.327	1.259	1.209	1.170	1.140	1.115	1.095	1.074

## Appendix C

Graphical SCF results for various weld angles *θ* for a cruciform joint subjected to tensile, bending, and shearing loads.
